# Effect of an Artificial Intelligence–Based Self-Management App on Musculoskeletal Health in Patients With Neck and/or Low Back Pain Referred to Specialist Care: A Randomized Clinical Trial

**DOI:** 10.1001/jamanetworkopen.2023.20400

**Published:** 2023-06-27

**Authors:** Anna Marcuzzi, Anne Lovise Nordstoga, Kerstin Bach, Lene Aasdahl, Tom Ivar Lund Nilsen, Ellen Marie Bardal, Nora Østbø Boldermo, Gro Falkener Bertheussen, Gunn Hege Marchand, Sigmund Gismervik, Paul Jarle Mork

**Affiliations:** 1 Department of Public Health and Nursing, Norwegian University of Science and Technology, Trondheim, Norway; 2 Department of Physical Medicine and Rehabilitation, St Olavs Hospital, Trondheim, Norway; 3 Department of Neuromedicine and Movement Science, Norwegian University of Science and Technology, Trondheim, Norway; 4 Department of Computer Science, Norwegian University of Science and Technology, Trondheim, Norway; 5 Unicare Helsefort Rehabilitation Center, Rissa, Norway; 6 Clinic of Anesthesia and Intensive Care, St Olavs Hospital, Trondheim, Norway

## Abstract

**Importance:**

Self-management is a key element in the care of persistent neck and low back pain. Individually tailored self-management support delivered via a smartphone app in a specialist care setting has not been tested.

**Objective:**

To determine the effect of individually tailored self-management support delivered via an artificial intelligence–based app (SELFBACK) adjunct to usual care vs usual care alone or nontailored web-based self-management support (e-Help) on musculoskeletal health.

**Design, Setting, and Participants:**

This randomized clinical trial recruited adults 18 years or older with neck and/or low back pain who had been referred to and accepted on a waiting list for specialist care at a multidisciplinary hospital outpatient clinic for back, neck, and shoulder rehabilitation. Participants were enrolled from July 9, 2020, to April 29, 2021. Of 377 patients assessed for eligibility, 76 did not complete the baseline questionnaire, and 7 did not meet the eligibility criteria (ie, did not own a smartphone, were unable to take part in exercise, or had language barriers); the remaining 294 patients were included in the study and randomized to 3 parallel groups, with follow-up of 6 months.

**Interventions:**

Participants were randomly assigned to receive app-based individually tailored self-management support in addition to usual care (app group), web-based nontailored self-management support in addition to usual care (e-Help group), or usual care alone (usual care group).

**Main Outcomes and Measures:**

The primary outcome was change in musculoskeletal health measured by the Musculoskeletal Health Questionnaire (MSK-HQ) at 3 months. Secondary outcomes included change in musculoskeletal health measured by the MSK-HQ at 6 weeks and 6 months and pain-related disability, pain intensity, pain-related cognition, and health-related quality of life at 6 weeks, 3 months, and 6 months.

**Results:**

Among 294 participants (mean [SD] age, 50.6 [14.9] years; 173 women [58.8%]), 99 were randomized to the app group, 98 to the e-Help group, and 97 to the usual care group. At 3 months, 243 participants (82.7%) had complete data on the primary outcome. In the intention-to-treat analysis at 3 months, the adjusted mean difference in MSK-HQ score between the app and usual care groups was 0.62 points (95% CI, −1.66 to 2.90 points; *P* = .60). The adjusted mean difference between the app and e-Help groups was 1.08 points (95% CI, −1.24 to 3.41 points; *P* = .36).

**Conclusions and Relevance:**

In this randomized clinical trial, individually tailored self-management support delivered via an artificial intelligence–based app adjunct to usual care was not significantly more effective in improving musculoskeletal health than usual care alone or web-based nontailored self-management support in patients with neck and/or low back pain referred to specialist care. Further research is needed to investigate the utility of implementing digitally supported self-management interventions in the specialist care setting and to identify instruments that capture changes in self-management behavior.

**Trial Registration:**

ClinicalTrials.gov Identifier: NCT04463043

## Key Points

**Question:**

What is the effect of an artificial intelligence–based smartphone app vs usual care alone or web-based self-management support on musculoskeletal health in patients with neck and/or low back pain referred to specialist care?

**Findings:**

In this randomized clinical trial of 294 adults with neck and/or low back pain referred to specialist care, the individually tailored app adjunct to usual care did not significantly improve musculoskeletal health more than usual care alone or nontailored web-based support at 3 months.

**Meaning:**

These findings suggest that further research is needed to investigate the utility of implementing digitally supported self-management interventions for those with neck and/or low back pain referred to specialist care and to identify instruments that capture changes in self-management behavior.

## Teaser

This randomized clinical trial assesses the effect of an artificial intelligence–based self-management app vs usual care alone or web-based self-management support on musculoskeletal health in adults with neck and/or low back pain referred to specialist care.

## Introduction

Neck pain and low back pain are among the leading causes of years lived with disability.^1^ In the US, back pain alone accounts for at least 264 million lost work days per year.^2^ Neck and low back pain are also among the main reasons to seek medical care,^3^ with outpatient specialist services accounting for a large proportion of the costs.^4^ Given the expected increase in the prevalence of neck and low back pain^5^ and the limited availability of health care resources, promoting effective self-management is key to mitigating the societal burden of these conditions.

Current best evidence suggests that self-management for persistent pain should be tailored to the patient’s individual needs and capabilities.^6,7,8,9^ Self-management for neck and low back pain is recommended to include education and reassurance along with regular exercise and advice to maintain daily activities.^8,9,10,11^ However, adherence to self-management recommendations without feedback or reinforcement is challenging for most patients.^12,13^ Digital tools, such as smartphone apps and web-based resources, can provide patients with self-management support.^14,15^ An apparent strength of using digital tools in self-management interventions is their accessibility and possible implementation across care pathways. However, knowledge of their applicability and effects in the specialist care setting is lacking. Specialist care is characterized by high service demands and limited capacity, which can result in long waiting times for first consultations; thus, implementing evidence-based self-management digital tools at the time of referral might improve quality of care and patient outcomes in this setting.

A previous study^16^ recently evaluated the effectiveness of a knowledge-based artificial intelligence (AI) decision support system entitled SELFBACK, which supports individually tailored and evidence-based self-management of low back pain via a smartphone app. Results of a randomized clinical trial (RCT)^17^ indicated a small but favorable effect of the app-based intervention compared with usual care on pain-related disability among patients receiving primary care.^17,18^ For the current RCT, we adapted the content of the app to also target patients with neck pain.^19^ In addition, we developed a web-based self-management intervention without individual tailoring (e-Help)^19^ to serve as an active control condition.

The primary aim of this RCT was to determine the effect of the app adjunct to usual care vs usual care alone on musculoskeletal health in patients with neck and/or low back pain referred to specialist care. The secondary aim was to determine the effect of the app on musculoskeletal health compared with the e-Help intervention.

## Methods

### Study Design, Setting, and Participants

The trial was approved by the Norwegian Medicines Agency and the regional ethics committee in Norway. The trial protocol (Supplement 1) was preregistered on ClinicalTrials.gov and published.^19^ All participants provided written informed consent before entering the trial. The trial followed the Consolidated Standards of Reporting Trials (CONSORT) guideline for RCTs.^20^

We recruited adults 18 years or older with neck and/or low back pain who had been referred and accepted to the multidisciplinary outpatient clinic for back, neck, and shoulder rehabilitation at St. Olav’s Hospital in Trondheim, Norway. Inclusion criteria included owning a smartphone (iOS or Android operating system) with internet access and a working email address. Exclusion criteria included warning signs indicating possible cancer, fracture, cauda equina syndrome, infection, or other conditions prioritized for urgent treatment or examination; inability to take part in exercise or physical activity (eg, nonambulatory status, use of walking aids, and inability to get up and down from the floor independently); inability to speak and/or read the Norwegian language; or enrollment in the ongoing SELFBACK trial in the primary care setting.

All patients with neck and/or low back pain who were referred to and accepted on a waiting list for the outpatient clinic between July 9, 2020, and April 29, 2021, were invited to participate. Administrative staff at the clinic identified potential participants based on the information available at referral. Potential participants were sent an invitation text message with a link to a registration form containing information about the trial, eligibility questions, and a digital consent form. Patients who consented and fulfilled the eligibility criteria were invited by email to complete the online baseline questionnaire. Those unable or unwilling to complete the baseline questionnaire were excluded from the study. Of 377 patients assessed for eligibility, 76 did not complete the baseline questionnaire, and 7 did not meet the eligibility criteria; the remaining 294 patients were included in the study.

### Randomization

After completing the baseline questionnaire, participants were randomized to receive (1) the app-based intervention adjunct to usual care (app group), (2) the e-Help web-based intervention adjunct to usual care (e-Help group), or (3) usual care only (usual care group). Randomization was performed using a web-based trial management system administered by the Unit of Applied Clinical Research at the Norwegian University of Science and Technology. Participants were randomized using permuted blocks with random sizes from 4 to 20 and 1:1:1 allocation. To ensure concealed allocation, the block sizes were not disclosed to study personnel, and the randomization code was automatically generated by the trial system and released after participants were enrolled in the trial. After randomization, participants were informed about their group allocation via a text message (ie, participants were not blinded to group allocation).

### Intervention and Control Conditions

Participants in all 3 groups were instructed to follow any diagnostic- or treatment-related pathways as indicated by health care practitioners they may have consulted during the study period. At the first consultation at the clinic (approximately 6-8 weeks after referral), all patients received usual care, which comprised a clinical examination and the offer of suitable treatment in accordance with current evidence-based guidelines. Treatment options included no further treatment, adjusted recommendations for primary care treatment, outpatient multimodal rehabilitation (individual and/or group sessions), or referral for surgical treatment.

Participants randomized to the app group were instructed to install the app via a link provided in the text message containing the information about group allocation. Correspondingly, participants randomized to the e-Help group received a link to the website and login instructions (ie, the website was accessible only for participants randomized to receive the e-Help intervention). Participants randomized to the intervention groups had unrestricted access to the app or e-Help website throughout the study period.

The app provides participants with weekly and individually tailored self-management recommendations for physical activity, strength and flexibility exercises, and daily educational messages as well as access to different tools and resources (goal setting, mindfulness audio files, pain-relieving exercises, and sleep reminders) that participants could use at their convenience (eFigure in Supplement 2). The development of the app has been described in detail elsewhere.^16,17,18,19,21^

Case-based reasoning is a knowledge-based AI method^22^ used within the app system to tailor recommendations by reusing previous similar and successful cases.^23^ This method enables patient-centered recommendations based on what has or has not been successful in previous patients. To personalize the self-management support, the system uses weekly reports and information collected via the app (ie, symptom progression, completion of exercises, barriers to self-management, and physical activity [number of steps collected] via the smartphone) along with the latest questionnaire information. By following the weekly recommendations, patients could collect badges and rewards displayed in the app. Moreover, push notifications triggered by the patient’s self-management behavior (eg, completion of exercises) were sent via the app to motivate and reinforce the desired self-management behavior.

The e-Help website offered evidence-based support and advice on self-management of neck and low back pain. Participants could access the website via their own device (eg, personal computer, smartphone, or tablet) at any time throughout the study period. The website has 4 main sections: (1) a home page, which describes the purpose of the e-Help intervention and how to use the e-Help resources; (2) educational messages; (3) videos and descriptions of strength and flexibility exercises; and (4) additional resources and educational tools, including instructions on goal setting, pacing techniques, sleep hygiene, and mindfulness along with practice audio files and links to relevant external websites. The content in sections 2 to 4 imitates the content in the app (ie, the same assortment of exercises); however, individual tailoring is not provided on the e-Help website. Clinicians at the outpatient clinic (including N.Ø.B., G.F.B., G.H.M. and S.G.) and a patient representative were involved in the development of the website to ensure it aligned with treatment principles at the clinic.

### Outcomes and Follow-up

Sociodemographic information was collected at baseline. Outcomes were assessed using an online questionnaire at baseline, 6 weeks, 3 months, and 6 months. The primary outcome was the mean difference in Musculoskeletal Health Questionnaire^24^ (MSK-HQ) scores between groups at 3 months. The Norwegian version of the MSK-HQ is a reliable and valid measure of musculoskeletal health among people on sick leave due to a musculoskeletal disorder.^25^ The MSK-HQ scale ranges from 0 to 56 points, with higher scores indicating better musculoskeletal health. In addition, we examined the difference in the proportion of patients reporting a 4-point or higher improvement in MSK-HQ score.

Secondary outcomes included low back pain–related disability assessed by the Roland-Morris Disability Questionnaire (range, 0-24, with higher scores indicating more pain-related disability)^26^; neck pain–related disability assessed by the Neck Disability Index (range, 0-50, with higher scores indicating more pain-related disability)^27^; mean and worst pain intensity in the preceding week rated on a numerical rating scale (range, 0-10, with higher scores indicating higher intensity)^28^; confidence in ability to cope despite pain assessed by the Pain Self-Efficacy Questionnaire (range, 0-60, with higher scores indicating greater confidence)^29^; cognitive and emotional perceptions of illness assessed by the Brief Illness Perception Questionnaire (range, 0-80, with higher scores indicating greater illness perception)^30^; health-related quality of life assessed by the EuroQol 5-dimension questionnaire, weighted according to the Danish value set^31^ (range, 0-1, with higher scores indicating better health status) and the EuroQol visual analog scale (range, 0-100, with higher scores indicating better health status)^32^; and overall improvement assessed by the Global Perceived Effect scale (range, −5 to 5, with positive scores indicating improvement of pain [anchor response of *very much better*] and negative scores indicate worsening of pain [anchor response of *very much worse*]).^33^ Additional exploratory outcomes were also evaluated as prespecified in the statistical analysis plan.^34^

### Adverse Events

Adverse events were defined as events causing participants to consult a health care professional due to circumstances potentially related to the interventions. Occurrences of adverse events were registered with the Norwegian health authorities when participants reported them to study personnel.

### Statistical Analysis

The planned sample size of at least 279 participants (93 in each group) was based on a power of 90% to detect a 4-point mean group difference in MSK-HQ score at 3 months, assuming an SD of 10 points, a correlation of 0.4 between repeated measures in the same participants, 2-sided α = .05, and a 30% study withdrawal rate during follow-up.^19^

The primary intention-to-treat analysis estimated mean group differences (with 95% CIs) in MSK-HQ score at 3 months using a constrained longitudinal data analysis approach fit with a linear mixed model.^35^ In this analysis, baseline and all follow-up values were modeled as dependent variables, and mean baseline values were constrained to be equal in the 3 groups, thereby accounting for any random differences in the outcome variable at baseline. To account for the dependency in observations within participants over time, the linear mixed model included a random intercept for each participant.

All effect estimates were adjusted for potentially important estimators of the outcome, including age (years), sex (male or female), educational level (<10 years, 10-12 years, or >12 years), and baseline mean pain intensity in the past week (on a scale of 0-10). Preplanned sensitivity analyses of the primary outcome included (1) multiple imputation of missing values using a multivariate normal approach and 20 imputed data sets, (2) complete case analysis including participants with data for all time points, and (3) per-protocol analysis of participants who adhered to the intervention.^34^ The per-protocol analysis included 2 approaches. In the first approach, adherence was defined as having accessed the intervention at least once (for both intervention groups); in the second approach, adherence was defined as having generated 6 or more self-management plans during the first 12 weeks after randomization (for the app group only). Assumptions related to the normality and homogeneity of residuals and the normality of random intercepts were assessed for all models. Analysis of mean group difference at 6 months and analyses of secondary and exploratory outcomes followed the same analytic approach used for the primary analysis.

We estimated the risk ratio for a 4-point or greater improvement in MSK-HQ score from baseline to 3-month follow-up using a Poisson generalized estimated equation (GEE) model. Similar GEE analyses were performed to estimate odds ratios for secondary binary outcomes using a logistic model. All GEE models assumed an exchangeable correlation structure with a robust variance estimator.

All estimates of precision were based on 2-sided tests. Statistical significance was defined as 2-tailed *P* < .05. Analyses were performed by a blinded researcher (T.I.L.N.) using Stata software, version 17 (StataCorp LLC),^36^ and the interpretation of blinded results was published.^37^

## Results

The flow of participants through the trial is shown in the Figure. In total, 294 participants were randomized; of those, 99 were in the app group, 98 were in the e-Help group, and 97 were in the usual care group. Overall, the mean (SD) age was 50.6 (14.9) years, and the mean (SD) body mass index (calculated as weight in kilograms divided by height in meters squared) was 26.8 (4.2); 173 participants (58.8%) were women, 121 (41.2%) were men, and 158 (53.7%) had full-time employment. Among the 99 participants randomized to the app group, 70 (70.7%) downloaded the app and generated at least 1 self-management plan, while 29 (29.3%) never accessed the app. Correspondingly, among the 98 participants randomized to the e-Help group, 90 (91.8%) accessed the website at least once. Among all participants, complete MSK-HQ data were obtained from 228 patients (77.6%) at 6 weeks, 243 (82.7%) at 3 months, and 173 (58.8%) at 6 months. Baseline characteristics were similar between the groups (Table 1).

**Figure.**
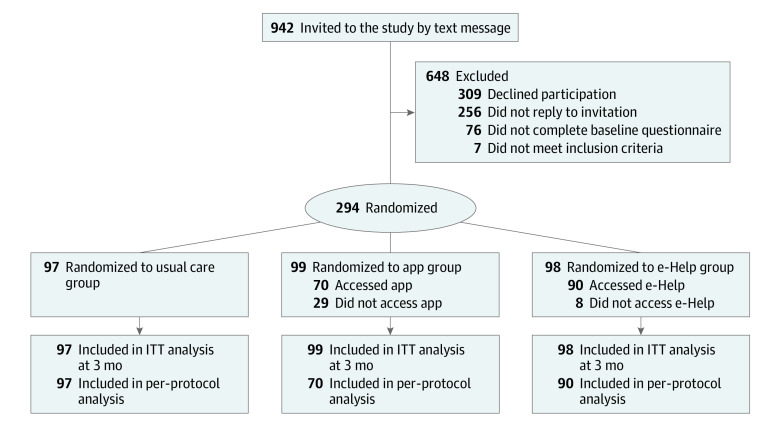
Study Flowchart Five participants in the usual care group withdrew from the study; of those, 3 participants did not report any reason, 1 did not benefit from the study, and 1 had pain elsewhere. Eighteen participants in the app group withdrew from the study; of those, 5 participants did not report any reason, 3 had technical problems, 3 found the app not relevant, 2 thought that joining the study was a prerequisite to get treatment at the clinic, 2 had other health issues, 2 had pain elsewhere, and 1 had personal reasons. Eleven participants in the e-Help group withdrew from the study; of those, 6 did not report any reason, 2 changed clinic, 1 had pain elsewhere, 1 thought it was too much effort, and 1 had a change in clinical situation and found no meaning in participating any longer. The main per-protocol analysis was based on accessing the intervention at least once for both the app and e-Help intervention groups. ITT indicates intention to treat.

**Table 1.** Baseline Characteristics of Study Participants

Characteristic	Participants, No. (%)			
	All (n = 294)	Usual care group (n = 97)^a^	App group (n = 99)^b^	e-Help group (n = 98)^c^
**Sociodemographic characteristics**				
Age, mean (SD) [range], y	50.6 (14.9) [18-86]	51.0 (15.2) [18-84]	50.3 (14.3) [18-80]	50.4 (15.1) [20-86]
BMI, mean (SD) [range]	26.8 (4.2) [17-41]	26.7 (4.2) [19-40]	26.9 (4.0) [19-39]	26.8 (4.4) [17-40]
Sex				
Female	173 (58.8)	58 (59.8)	60 (60.6)	55 (56.1)
Male	121 (41.2)	39 (40.2)	39 (39.4)	43 (43.9)
Educational level, y				
<10	26 (8.8)	7 (7.2)	10 (10.1)	9 (9.2)
10-12	88 (29.9)	31 (32.0)	28 (28.3)	29 (29.6)
>12	180 (61.2)	59 (60.8)	61 (61.6)	60 (61.2)
Full-time employment	158 (53.7)	47 (48.5)	57 (57.6)	54 (55.1)
Married or living with partner	221 (75.2)	72 (74.2)	72 (72.7)	77 (78.6)
**Neck and low back pain history**				
Pain localization				
Low back	166 (56.5)	53 (54.6)	52 (52.5)	61 (62.2)
Neck	46 (15.6)	13 (13.4)	18 (18.2)	22 (22.4)
Neck and low back	82 (27.9)	31 (32.0)	29 (29.3)	15 (15.3)
Days with pain in past year				
0	2 (0.7)	1 (1.0)	1 (1.0)	0
1-7	5 (1.7)	1 (1.0)	1 (1.0)	3 (3.1)
8-30	16 (5.4)	7 (7.2)	5 (5.1)	4 (4.1)
>30 but not every day	89 (30.3)	24 (24.7)	38 (38.4)	27 (27.6)
Every day	182 (61.9)	64 (66.0)	54 (54.5)	64 (65.3)
Use of pain medication, d/wk				
0	93 (31.6)	36 (37.1)	28 (28.3)	29 (29.6)
1-2	69 (23.5)	17 (17.5)	20 (20.2)	32 (32.7)
3-5	61 (20.7)	21 (21.6)	22 (22.2)	18 (18.4)
Every day	71 (24.1)	23 (23.7)	29 (29.3)	19 (19.4)
**Baseline measure of primary outcome**				
Musculoskeletal Health Questionnaire, mean (SD)^d^	29.2 (8.5)	29.0 (9.0)	30.0 (9.1)	28.5 (7.1)
**Baseline measures of secondary outcomes**				
Roland-Morris Disability Questionnaire, mean (SD)^e^^,^^f^	10.9 (5.1)	11.1 (5.0)	10.1 (5.3)	11.5 (4.9)
Neck Disability Index, mean (SD)^g^^,^^h^	18.3 (7.0)	17.8 (6.7)	18.1 (7.8)	19.1 (6.0)
Mean pain intensity in past wk (NRS), mean (SD)^i^	5.4 (1.8)	5.4 (1.8)	5.3 (2.0)	5.6 (1.6)
Worst pain intensity in past wk (NRS), mean (SD)^i^	7.0 (1.8)	6.9 (1.9)	6.8 (2.0)	7.2 (1.5)
EQ-VAS, mean (SD)^j^	53.0 (18.6)	50.7 (19.3)	55.7 (18.0)	52.5 (18.2)
EQ-5D (weighted), mean (SD)^k^	0.64 (0.15)	0.63 (0.14)	0.65 (0.17)	0.63 (0.14)
Pain Self-Efficacy Questionnaire, mean (SD)^l^	36.8 (13.3)	36.9 (13.1)	38.0 (13.1)	35.5 (13.6)
Brief Illness Perception Questionnaire, mean (SD)^m^	51.2 (9.2)	52.2 (8.4)	51.3 (9.4)	50.2 (9.7)

Abbreviations: BMI, body mass index (calculated as weight in kilograms divided by height in meters squared); EQ-5D, EuroQoL 5-dimension questionnaire; EQ-VAS, EuroQol visual analog scale; NRS, numerical rating scale.

^a^ The usual care group received usual care only. Usual care comprised a clinical examination and an offer of suitable treatment in accordance with current evidence-based guidelines.

^b^ The app group received individually tailored app-delivered self-management support in addition to usual care.

^c^ The e-Help group received nontailored web-based self-management support in addition to usual care.

^d^ Score range, 0 to 56, with higher scores indicating better musculoskeletal health.

^e^ Score range, 0 to 24, with higher scores indicating more pain-related disability.

^f^ Includes 248 participants with low back pain or neck and low back pain (84 in the usual care group, 81 in the app group, and 83 in the e-Help group).

^g^ Score range, 0 to 50, with higher scores indicating more pain-related disability.

^h^ Includes 128 participants with neck pain or neck and low back pain (44 in the usual care group, 47 in the app group, and 37 in the e-Help group).

^i^ Scale range, 0 to 10, with higher scores indicating higher intensity level of pain.

^j^ Scale range, 0 to 100, with higher scores indicating better health status.

^k^ Score range, 0 to 1, with higher scores indicating better health status.

^l^ Score range, 0 to 60, with higher scores indicating greater confidence.

^m^ Score range, 0 to 80, with higher scores indicating greater illness perception.

### Primary Outcome

From baseline to 3 months, the within-group mean change in MSK-HQ score was 4.6 points (95% CI, 2.9-6.3 points) for the usual care group, 5.4 points (95% CI, 3.7-7.2 points) for the app group, and 4.1 points (95% CI, 2.3-5.8 points) for the e-Help group. There were no statistically significant differences in MSK-HQ scores among participants in the app group compared with other groups. The adjusted mean MSK-HQ score at 3 months was 0.62 points (95% CI, −1.66 to 2.90 points; *P* = .60) higher in the app group compared with the usual care group and 1.08 points (95% CI, −1.24 to 3.41 points; *P* = .36) higher in the app group compared with the e-Help group (Table 2). The results were similar at 6 months (Table 2) and in sensitivity analyses (eTable 1 in Supplement 2).

**Table 2.** Mean Scores and Between-Group Differences in Primary and Secondary Outcomes at 3 Months and 6 Months

Outcome	Scores, mean (SD)^a^			Between-group differences, mean (95% CI)^e^	
	Usual care group (n = 97)^b^	App group (n = 99)^c^	e-Help group (n = 98)^d^	App vs usual care	App vs e-Help
**Primary**					
Musculoskeletal Health Questionnaire^f^					
Baseline^g^	29.2 (8.5)			NA	NA
3 mo	33.8 (10.1)	34.6 (10.2)	33.2 (11.0)	0.62 (−1.66 to 2.90)	1.08 (−1.24 to 3.41)
6 mo	35.5 (10.4)	36.4 (9.9)	33.4 (10.5)	0.48 (−2.22 to 3.18)	2.44 (−0.25 to 5.12)
**Secondary**					
Roland-Morris Disability Questionnaire^h^^,^^i^					
Baseline^g^	10.9 (5.1)			NA	NA
3 mo	9.3 (5.6)	8.8 (5.3)	9.4 (5.8)	−0.45 (−1.79 to 0.88)	−0.57 (−1.92 to 0.78)
6 mo	8.2 (6.1)	7.9 (5.4)	9.7 (6.1)	−0.17 (−1.71 to 1.38)	−1.64 (−3.17 to −0.13)
Neck Disability Index^j^^,^^k^					
Baseline^g^	18.3 (7.0)			NA	NA
3 mo	15.8 (6.6)	15.8 (7.4)	15.0 (7.1)	0.06 (−2.05 to 2.16)	0.78 (−1.49 to 3.04)
6 mo	14.6 (7.3)	16.0 (8.0)	15.2 (7.8)	1.57 (−0.95 to 4.10)	0.86 (−1.86 to 3.57)
Mean pain intensity in past wk (NRS)^l^					
Baseline^g^	5.4 (1.9)			NA	NA
3 mo	4.6 (2.1)	4.6 (2.2)	4.8 (2.2)	−0.05 (−0.59 to 0.48)	−0.22 (−0.77 to 0.33)
6 mo	4.4 (2.5)	4.1 (2.3)	4.7 (2.4)	−0.26 (−0.88 to 0.36)	−0.57 (−1.19 to 0.05)
Worst pain intensity in past wk (NRS)^l^					
Baseline^g^	7.0 (1.8)			NA	NA
3 mo	6.2 (2.3)	6.0 (2.4)	6.0 (2.4)	−0.20 (−0.78 to 0.37)	0.05 (−0.54 to 0.63)
6 mo	5.8 (2.7)	5.5 (2.5)	6.1 (2.6)	−0.34 (−1.01 to 0.33)	−0.66 (−1.32 to 0.01)
EQ-VAS^m^					
Baseline^g^	53.0 (18.6)			NA	NA
3 mo	57.1 (18.2)	58.8 (20.0)	56.6 (20.4)	1.63 (−3.08 to 6.34)	1.95 (−2.86 to 6.75)
6 mo	61.0 (19.3)	60.0 (20.3)	54.8 (20.2)	−1.37 (−6.86 to 4.12)	4.56 (−0.92 to 10.03)
EQ-5D (weighted)^n^					
Baseline^g^	0.64 (0.15)			NA	NA
3 mo	0.70 (0.14)	0.70 (0.15)	0.66 (0.20)	0.01 (−0.03 to 0.04)	0.04 (0 to 0.08)
6 mo	0.70 (0.16)	0.72 (0.13)	0.68 (0.17)	0.01 (−0.04 to 0.05)	0.02 (−0.02 to 0.07)
Pain Self-Efficacy Questionnaire^o^					
Baseline^g^	36.8 (13.3)			NA	NA
3 mo	41.2 (12.6)	41.0 (12.4)	38.8 (13.8)	−0.31 (−3.28 to 2.66)	1.98 (−1.05 to 5.01)
6 mo	43.0 (13.5)	42.2 (12.0)	36.9 (14.9)	−1.12 (−4.57 to 2.34)	4.83 (1.37 to 8.29)
Brief Illness Perception Questionnaire^p^					
Baseline^g^	45.6 (10.3)			NA	NA
3 mo	42.3 (11.0)	41.9 (11.0)	42.4 (14.1)	−0.25 (−2.91 to 2.40)	−0.30 (−3.00 to 2.40)
6 mo	40.6 (12.2)	39.7 (13.6)	43.8 (13.7)	−0.50 (−3.60 to 2.59)	−3.65 (−6.74 to −0.56)
Global Perceived Effect scale^q^					
Baseline^g^	NA			NA	NA
3 mo	0.25 (1.95)	1.02 (1.64)	0.70 (2.24)	0.74 (0.24 to 1.25)	0.28 (−0.23 to 0.80)
6 mo	1.01 (2.38)	1.02 (2.59)	0.53 (2.22)	−0.04 (−0.65 to 0.57)	0.44 (−1.16 to 1.05)

Abbreviations: EQ-5D, EuroQoL 5-dimension questionnaire; EQ-VAS, EuroQol visual analog scale; NA, not applicable; NRS, numerical rating scale.

^a^ Marginal means are from a crude linear mixed model and SDs are from raw data among persons with information at the specific time points.

^b^ The usual care group received usual care only. Usual care comprised a clinical examination and suitable treatment in accordance with current evidence-based guidelines. Treatment options included no further treatment, adjusted recommendations for primary care treatment, outpatient multimodal rehabilitation (individual and/or group sessions), or referral for surgical treatment.

^c^ The app group received individually tailored app-delivered self-management support in addition to usual care.

^d^ The e-Help group received nontailored web-based self-management support in addition to usual care.

^e^ Adjusted for age (years), sex (male or female), educational level (<10 years, 10-12 years, or >12 years), and mean pain intensity in the past week at baseline (continuous; scale range, 0-10, with higher scores indicating higher intensity level of pain).

^f^ Score range, 0 to 56, with higher scores indicating better musculoskeletal health.

^g^ Mean baseline values were constrained to be equal in the 3 groups, thereby accounting for any random differences in the outcome variable at baseline.

^h^ Score range, 0 to 24, with higher scores indicating more pain-related disability.

^i^ Values include only participants reporting low back pain only or neck and low back pain (n = 248).

^j^ Score range, 0 to 50, with higher scores indicating more pain-related disability.

^k^ Values include only participants reporting neck pain only or neck and low back pain (n = 128).

^l^ Scale range, 0 to 10, with higher scores indicating higher intensity level of pain.

^m^ Scale range, 0 to 100, with higher scores indicating better health status.

^n^ Score range, 0 to 1, with higher scores indicating better health status.

^o^ Score range, 0 to 60, with higher scores indicating greater confidence.

^p^ Score range, 0 to 80, with higher scores indicating greater illness perception.

^q^ Scale range, −5 to 5, with positive scores indicating improvement of pain (anchor response of *very much better*) and negative scores indicating worsening of pain (anchor response of *very much worse*).

The percentage of participants reporting an improvement of 4 points or greater on the MSK-HQ from baseline to 3 months was 44.2% (38 of 86 participants) in the usual care group, 59.0% (46 of 78 participants) in the app group, and 46.8% (37 of 79 participants) in the e-Help intervention group. These proportions corresponded to adjusted risk ratios for improvement of 1.35 (95% CI, 1.00-1.82; *P* = .05) in favor of the app group compared with the usual care group and 1.23 (95% CI, 0.92-1.65; *P* = .34) in favor of the app group compared with the e-Help group, although these results were not statistically significant (Table 3).

**Table 3.** Proportion of Participants Who Reported Improvement of 4 Points or Greater on the Musculoskeletal Health Questionnaire and Group Comparisons at 3-Month and 6-Month Follow-up

Follow-up time	Usual care group (n = 97)^a^		App group (n = 99)^b^		e-Help group (n = 98)^c^		Between-group differences, RR (95% CI)^d^	
	No. improved/total No. (%)	RR (95% CI)^b^	No. improved/total No. (%)	RR (95% CI)^b^	No. improved/total No. (%)	RR (95% CI)^b^	App vs usual care	App vs e-Help
3 mo	38/86 (44.2)	1.12 (0.84-1.48)	46/78 (59.0)	1.51 (1.09-2.08)	37/79 (46.8)	1.23 (0.86-1.75)	1.35 (1.00-1.82)	1.23 (0.92-1.65)
6 mo	31/59 (52.5)	1.39 (1.03-1.87)	32/53 (60.4)	1.63 (1.15-2.29)	31/61 (50.8)	1.33 (0.93-1.90)	1.17 (0.85-1.61)	1.22 (0.88-1.69)

Abbreviation: RR, risk ratio.

^a^ The usual care group received usual care only. Usual care comprised a clinical examination and suitable treatment in accordance with current evidence-based guidelines. Treatment options included no further treatment, adjusted recommendations for primary care treatment, outpatient multimodal rehabilitation (individual and/or group sessions), or referral for surgical treatment.

^b^ The app group received individually tailored app-delivered self-management support in addition to usual care.

^c^ The e-Help group received nontailored web-based self-management support in addition to usual care.

^d^ Adjusted for age (years), sex (male or female), educational level (<10 years, 10-12 years, or >12 years), and mean pain intensity in the past week at baseline (continuous; scale range, 0-10, with higher scores indicating higher intensity level of pain).

### Secondary Outcomes

None of the secondary outcomes differed between groups at 3 months except for the Global Perceived Effect score, which was significantly higher in the app group compared with the usual care group (mean difference, 0.74; 95% CI, 0.24-1.25; *P* = .004) (Table 2). At 6 months, there were no differences between the app group and the usual care group for any of the secondary outcomes (Table 2; eTables 2 and 3 in Supplement 2). Exploratory outcomes are reported in eTables 4 and 5 in Supplement 2.

### Adverse Events

One participant randomized to the app-based intervention reported hospitalization due to dizziness during participation in the trial. However, this event was deemed unlikely to be related to the intervention and was spontaneously resolved.

## Discussion

This RCT found that among patients referred and admitted to specialist care due to neck and/or low back pain, the receipt of evidence-based and individually tailored self-management support via an AI-based app adjunct to usual care did not result in significantly improved musculoskeletal health at 3 months compared with usual care alone. The results for the primary and secondary outcomes were similar at 6 months.

Multidisciplinary pain treatment in specialist care commonly includes elements intended to improve patients’ self-management skills.^38^ In this way, digital interventions can potentially be used to reinforce and support patients’ self-management. The results of this trial indicated that the app was not an effective treatment adjunct to usual care for patients with neck and/or low back pain referred to specialist care. We are not aware of other studies that have tested similar interventions for self-management in the specialist care setting. A previous RCT in the primary care setting^17^ found a small but favorable reduction in pain-related disability among people with low back pain receiving the app-based intervention adjunct to usual care compared with those receiving usual care alone. Several factors related to the trial designs may partly explain these contrasting results. For example, unlike the current trial, clinicians in the primary care trial^17^ were involved in the recruitment of participants. Some evidence suggests that endorsement by a clinician may be important to facilitate and reinforce patients’ engagement in self-management.^39^ The different onboarding procedure used (ie, personal assistance in installing the app in the previous trial^17^ vs text message with a link to app installation in the current trial) might also have influenced participant engagement with the app-based intervention, which was lower in the current trial. In addition, patients referred to specialist care might represent a group with more complex symptom profiles compared with those receiving primary care,^40^ which could potentially influence their ability to self-manage. The process evaluation conducted along with the trial will provide more detailed insight into barriers and facilitators related to the uptake and use of digital interventions in specialist care.^19^

### Strengths and Limitations

This study has several strengths. These strengths include the 3-group design ensuring an active comparison group; well-balanced baseline characteristics between groups, suggesting successful randomization; a high retention rate at the primary end point of 3 months; and a preplanned blinded analysis.

The study also has limitations. First, the uptake of the interventions was suboptimal (eg, 29.3% of those in the app group never accessed the app). Consistent with the pragmatic nature of the trial, participants were required to install the app themselves, but phone assistance was offered to those who needed it. This process might have influenced uptake of the app, particularly among participants with limited digital literacy. Low engagement with the intervention could explain the lack of effect on the primary outcome, even if the app itself was potentially effective. However, our per-protocol analyses did not support this notion. In addition, given the lack of consensus on outcome measures for self-management behavior,^41^ we used musculoskeletal health as a proxy measure. This measure might have been suboptimal for detecting changes in self-management behavior if they existed.

## Conclusions

In this RCT of patients with neck and/or low back pain referred to specialist care, individually tailored and evidence-based self-management support delivered via an AI-based app adjunct to usual care did not significantly improve musculoskeletal health at 3 months compared with usual care alone or web-based self-management support without individual tailoring. Future studies should investigate the utility of implementing digitally supported self-management interventions in the specialist care setting and identify instruments that capture changes in self-management behavior.

## Notes

Supplement 1.
Trial Protocol

Supplement 2. **eFigure.** Start Screen of the SELFBACK App (Left) and 3 Main Components of the Weekly Self-Management Plan (Physical Activity [Steps], Exercise, and Education) **eTable 1.** Mean (SD) and Between-Group Differences in Primary Outcome at 3 Months for All Sensitivity Analyses **eTable 2.** Proportion of Participants Who Reported Improvement and Group Comparisons at 3-Month and 6-Month Follow-up for Disability Outcomes **eTable 3.** Odds Ratios for Secondary Binary Outcomes Comparing Groups at 3 Months and 6 Months **eTable 4.** Mean (SD) and Between-Group Differences for Exploratory Outcome at 3 Months and 6 Months **eTable 5.** Odds Ratios for Exploratory Binary Outcomes Comparing Groups at 3 Months and 6 Months

Supplement 3.
Data Sharing Statement
