# Supplementary material for: Effect of an Artificial Intelligence–Based Self-Management App on Musculoskeletal Health in Patients With Neck and/or Low Back Pain Referred to Specialist Care: A Randomized Clinical Trial
Source: JAMA Netw Open. 2023 Jun 27;6(6):e2320400. doi: 10.1001/jamanetworkopen.2023.20400 (PMC10300712; doi:10.1001/jamanetworkopen.2023.20400)
Supplement: Supplement 1. — Trial Protocol [file jamanetwopen-e2320400-s001.pdf]

Supplement 1. Trial protocol and Statistical Analysis Plan

The effectiveness of a self-management app and an e-Help webpage in people with low back and neck pain on a waiting list for outpatient rehabilitation: a protocol for a randomized controlled trial

Trial registration

The trial will be registered in the database at www.clinicaltrials.gov.

Protocol version: 1.0 (23/04/2020)

Funding

Funding to undertake this trial has been obtained through a grant from the European Union Horizon 2020 Research & Innovation Program (Grant no. 777090) as well as internal project funding.

This protocol was developed in accordance to the Standard Protocol Items: Recommendation for Intervention Trials (SPIRIT 2013) Statement [1] and the Consolidated Standards of Reporting Trials (CONSORT 2010) guidelines [2].

1. Introduction

Low back pain (LBP) and neck pain (NP) are highly prevalent musculoskeletal conditions [3, 4] and the leading cause of years lived with disability globally [5]. LBP and NP not only result in high personal suffering but also present as a major societal challenge due to high economic costs associated with reduced work productivity, sickness absence and greater health care resource utilization [6, 7]. As the global burden of LBP and NP is expected to increase in the coming years [8, 9], the identification of more effective and cost-effective strategies for the management of LBP and NP are the current priorities [10].

The use of eHealth and mHealth solutions for management of chronic pain can be a promising approach as they are easy to deliver, inexpensive and safe. In addition, mHealth solutions have the possibility to provide *tailored* support to individual patients which might be more beneficial compared to non-tailoring approaches [11, 12]. Although many smartphone apps for self-management of LBP are available, most of them are of poor quality and their effectiveness on pain and functional outcomes have not been documented [13, 14]. In an EU-funded project, we have developed an evidence-based decision support system (DSS) – SELFBACK app – designed to support and reinforce self-management of LBP [15]. The effectiveness of SELFBACK for supporting self-management of LBP among patients in primary care is currently under investigation. Here, we outline a protocol for testing a version of the SELFBACK app that is adopted to target both LBP and NP in a secondary care setting. The effectiveness of SELFBACK will be compared to a non-tailoring web-based solution – eHelp - providing evidence-based self-management content equivalent to the SELFBACK and to usual care only.

1.1 Objectives and hypothesis

The objective of the trial is to compare the effectiveness of the SELFBACK app in addition to usual care (intervention group 1) to the e-Help webpage in addition to usual care (intervention group 2) and to usual care only (control group) in terms of musculoskeletal health status measured by the Musculoskeletal Health Questionnaire (MSK-HQ) among patients with LBP and/or NP on a waiting list for outpatient rehabilitation.

The first hypothesis is that patients with LBP and/or NP randomized to receive the SELFBACK intervention would show greater improvements in health status at 3-month follow up compared to those randomized to usual care only. The second hypothesis is that the SELFBACK intervention would be superior to the e-Help webpage due to the additional tailoring of self-management program.

2. Methods

This study will be conducted according to the principles of the Declaration of Helsinki (64th WMA General Assembly, Fortaleza, Brazil, October 2013). For the SELFBACK app intervention, we have notified the Norwegian Medicines Agency (SLV) and asked for approval of compliance with the relevant national regulations and EU Guidelines on Medical Software Devices.

2.1 Trial design

This is a single-blinded superiority randomized controlled trial (RCT) with three parallel groups. Participants with LBP and/or NP on a waiting list for a rehabilitation program will be randomized to receive: 1) the SELFBACK intervention in addition to usual care; 2) the e-Help webpage in addition to usual care; or 3) usual care only.

2.2 Study setting

Patients on waiting list for treatment at the multidisciplinary outpatient clinic for back, neck and shoulder rehabilitation, St. Olavs Hospital, Trondheim University Hospital, Norway, due to LBP and/or NP will be invited to this study.

2.3 Eligibility criteria

2.3.1 Inclusion criteria

- Adults ≥ 18 years
- On waiting list for treatment at the multidisciplinary outpatient clinic for back, neck and shoulder rehabilitation, St. Olavs Hospital, Trondheim University Hospital, Norway, due to LBP and/or NP
- Own and use a smartphone with internet access to download the mobile application
- Able to provide consent (i.e., not reduced ability to give consent)

2.3.2 Exclusion criteria

- Patients with less than 4 weeks waiting time until scheduled appointment at clinic (i.e., patients prioritized for urgent treatment/examination)
- Unable to take part in exercise/physical activity, e.g. non-ambulatory patients, use of walking aids, unable to get up and down the floor independently
- Unable to speak and/or read Norwegian

2.4 Recruitment of study participants

Recruitment will take place through the multidisciplinary outpatient clinic for back, neck, and shoulder rehabilitation at St. Olavs Hospital, Trondheim. The clinic receives approximately 4000-4500 referrals for LBP and/or NP each year (about 350 referrals/month) and the majority are received from general practitioners. Referrals from other primary and secondary care sources may also occur. Referred patients are registered into the system by a secretary and then assessed for suitability to undertake a rehabilitation program by a chief physician at the clinic. Around 49% of patients are admitted to the rehabilitation program based on referral and put on a waiting list. The remaining 51% are not admitted and either 1) referred to another specialist, e.g. orthopedic, surgeon, pain specialist (around 27%), or 2) referred to primary care provider (around 24%). Recruitment of participants for this trial is expected to start in May 2020 and end by December 2020.

2.5 Identification and screening of participants

Patients who are admitted to rehabilitation *and* currently on a waiting list will be the target group for this trial. Potential participants will be identified based on their allocation to the LBP and NP rehabilitation programs. To patients admitted to treatment, the clinic will send a letter within a week after the decision on admission. In parallel with sending the letter from the clinic, an SMS will be sent to potential participants with a link to a registration form. A phone number of a researcher will be provided in the SMS so that patients can call if they have any questions about the study before accessing the registration form. The registration form is accessed by participants through the eForsk platform via BankID and contains further information about the study with contact details of a researcher, self-assessment eligibility questions, the self-reported Fibromyalgia Survey Criteria and the StarT MSK stratification tool, and the participant information consent form. Participants who are not eligible to take part of the study will not be shown the informed consent process but will be asked if they consent

for the data collected to be used for quality assurance purposes at the clinic. If they do not consent, the data will be erased.

2.6 Informed consent process and enrolment of participants

If eligible and interested to participate, potential participants will be required to provide digital informed consent via the eForsk platform or written informed consent (see below). Potential participants can leave the registration form and return to it at a later stage should they need more time to consider their participation. At any time during this process, they will have the opportunity to contact a researcher who will answer any questions or concerns they might have about the study and their participation.

One reminder SMS will be sent to potential participants after a minimum of 3 days with the link to the registration form. If no response is registered after another additional 3 days, the potential participants will be contacted via phone by a secretary/research assistant at the clinic and asked whether they are interested in the study and/or assist participants if they encountered any technical issues with accessing the registration form. An option to perform the eligibility screening via phone will be provided for those who indicate this preference as well as the informed consent form, i.e. digital or hard copy formats will be sent to them accordingly to facilitate timely enrolment into the trial.

The following options will be available to participants should they choose the written consent form:

1) Send a photo of the signed consent form by SMS
2) Send a copy of the scanned signed consent by email
3) Send a hard copy of the signed consent form by mail

Once the consent process is completed, a link to the baseline questionnaire will be provided to participants by email. If the questionnaire is not filled out within 3 days, a reminder will be sent to them to avoid delays in the enrolment of the trial. If no response is registered after 5 days, the participant will be considered withdrawn from the study. A participant number will be assigned to those who complete the baseline questionnaire and subsequently the randomization will be performed online via a web-based randomization system.

2.7 Randomization

Participants are randomized to receive 1) SELFBACK intervention in addition to usual care; 2) e-Help webpage in addition to usual care; or 2) usual care only. Randomization is performed as block randomization with permuted blocks of random size (10 to 20 participants). Randomization will be performed by a web-based program (WebCRF) administered by the Unit of Applied Clinical Research, Faculty of Medicine and Health Sciences, Norwegian University of Science and Technology. This unit is not otherwise involved in the trial management or study conduct. The allocation ratio is balanced between the three groups, i.e., the SELFBACK intervention, e-Help webpage and the control group. The participants’ flow is described in **Figure 1**.

2.8 Blinding

This study is a single-blinded trial. Participants and health personnel at the clinic will not be blinded to group allocation, whereas researchers performing the analysis and the interpretation of the results will be blinded to group allocation. Once the study is completed, the data will be extracted from the database in anonymized form for statistical analyses, i.e. all personal information that may identify specific participants or group allocation will be removed and the intervention and control groups will be

randomly labelled as A, B and C. The randomization key (document entailing information on which group is which) is kept at the Unit of Applied Clinical Research at NTNU. They will provide the randomization key to the research team once a blinded interpretation of the results is finalized.

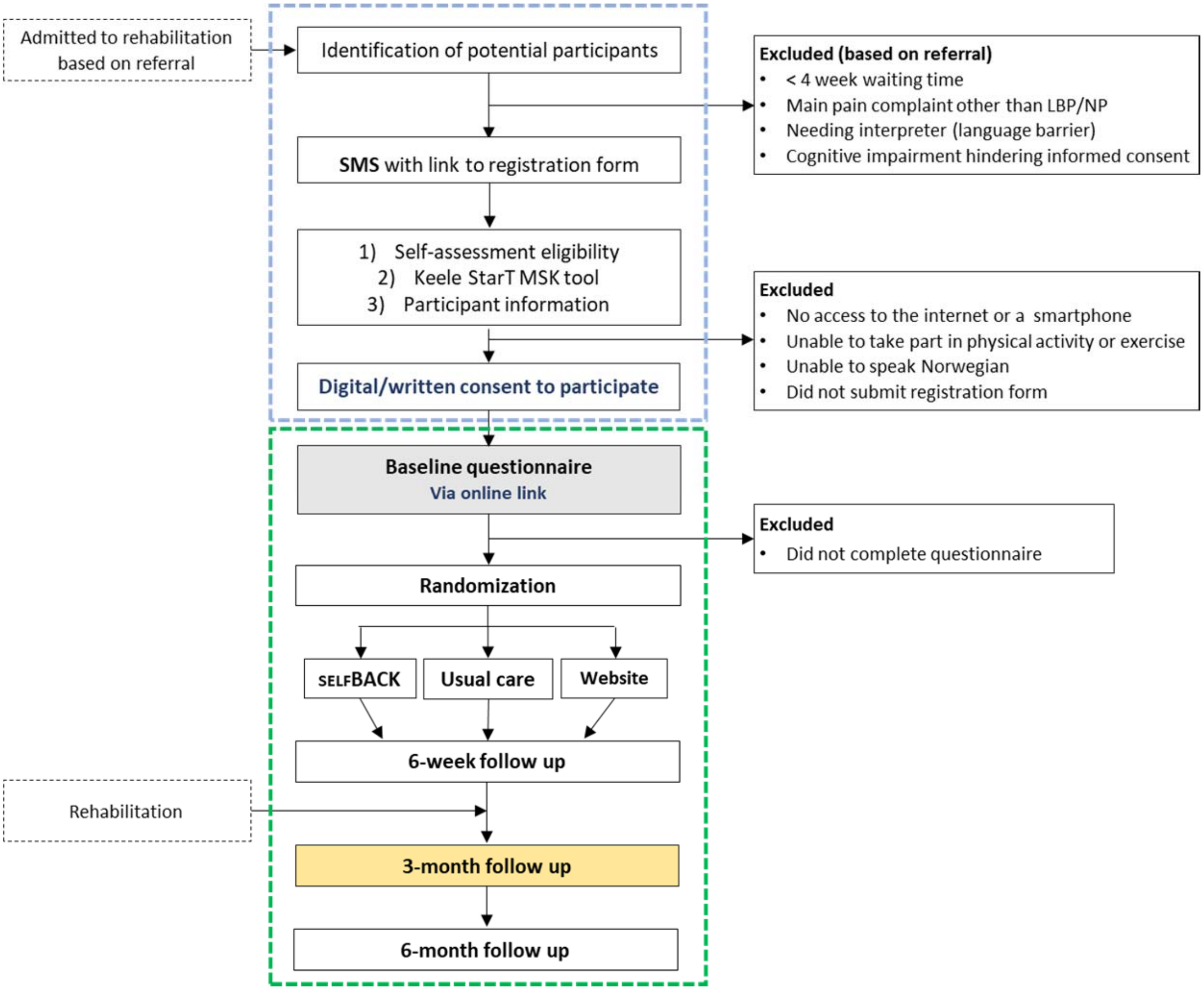

Figure 1 Study procedure and participant flow through the trial. Blue dotted box represents the actions taken by staff at the clinic; green dotted box represents the actions taken by researchers.

3. Interventions

3.1 Usual care (control group)

Participants randomized to usual care will follow any diagnostic or treatment-related pathways as chosen by the health care practitioner (HCP) they *may* consult. They will be allowed to seek care and receive treatments or help elsewhere as normal. Follow up assessments for the trial will be performed at pre-defined time points (see section 4).

3.2 SELFBACK app in addition to usual care (intervention group 1)

Patients randomized to the SELFBACK group will receive an SMS with link to the app that automatically install the app. The SELFBACK intervention is a DSS designed to support self-management of LBP and NP, delivered to participants in the form of the SELFBACK app. The SELFBACK app provides an individually tailored self-management plan to participants by matching the participant's health information with targeted educational messages, physical activity advices and exercise recommendations via the DSS. The intervention is not intended to replace follow-up by HCP, but to supplement the HCP's care and the participant is informed accordingly. Therefore, participants randomized to the SELFBACK intervention may continue to seek care, treatment or help elsewhere as normal.

The SELFBACK system constitutes a data-driven predictive DSS that uses the Case-Based Reasoning (CBR) methodology [16, 17] to capture and reuse participant cases in order to suggest the most suitable self-management plan for new participants. The data sources for the CBR system comprise: 1) the initial participant data collected by the baseline web-based questionnaire, and 2) a weekly report by the participant in the SELFBACK app (including pain, function, fear-avoidance, workability, sleep, self-efficacy, stress, health belief and barriers). On a *weekly* basis, this information is used to revise the self-management plan by matching the characteristics of the current participant case with existing successful participant cases in the SELFBACK case-base. The weekly tailoring questions will only be given if relevant for participants. Consequently, the DSS will deliver an individualized self-management plan for the coming week via the SELFBACK app. A full description of the DSS is published elsewhere [15]. Importantly, all interaction between the participant and the SELFBACK DSS happens via the SELFBACK app. There is no interaction between the DSS and HCPs.

3.2.1 The SELFBACK self-management content

The DSS builds the weekly self-management plan from three types of content:

1) Physical activity level (i.e., number of steps) and goals

    The SELFBACK app prompts participants to set a goal for physical activity by suggesting a gradual increase in daily steps if the past week's goal was achieved. A 10% increase is suggested, until a goal of 10.000 steps per day is reached. Participants may adjust the suggested goal, before accepting it. During the week, participants can see the achieved step-count per day and track their progress. The lowest step count goal that is possible to set will be 3000 steps per day and this was chosen to reflect the functional disability in the participant group in the trial which may also affect their physical activity level. Based on the achieved daily step count from previous week, the step count goal for the coming week is adjusted, and educational messages and notifications aimed to motivate more physical activity is pushed to the participant through the app.

2) Strength and flexibility exercises

The exercise material is compiled of exercises organized in different targets, e.g. back-, neck-, abdominal-, gluteal-, core muscle strength, pain relieve and flexibility. Participants are given an individualized exercise program with the default recommendation to perform exercises in 3-5 sessions per week of 15 minutes (i.e. three exercises with an estimated duration of 5 min per exercise). The number of exercises is adjusted according to participants' availability and to the anticipated level of difficulty defined by baseline questionnaire. If participants present with an acute pain flare-up or high pain ratings, they will be only offered pain-relieving exercises until an acceptable pain level is achieved. The exercises are presented to participants as a short video accompanied by written instructions that include recommendations on number of sets and repetitions. Participants will be prompted in the app to report completed number of sets and repetitions per exercise. Once participants report the volume for an individual exercise, the DSS will offer a new exercise at a more difficult level in the coming week's self-management plan. Likewise, the system will recommend an exercise at an easier level if a low level of completion is registered. In addition, participants can request new exercises (at the same level of difficulty and within the same group of exercises) at the end of the self-management plan if they experience problems completing the suggested exercise.

3) Educational material

The educational material is compiled of 14 main components ("information about LBP/NP", "understanding mind-body", "self-management for LBP/NP", "thoughts, behavior, attitude and feeling", "fitting in self-management in a busy life", "first aid when your back hurts", "LBP/NP and comorbidities", "goal-setting and action planning", "pacing and graded activity", "problem solving", "relaxation", "sleep and LBP/NP", "social support" and "overcoming barriers for self-management of LBP/NP"). For each main component, a tree-structure of educational messages has been created. Every short message is about 140 characters long. Some messages may include links to longer, more explanatory text (max 500 characters) or tools that can be used to help with self-managing LBP/NP, e.g. goal setting tool, sleep advice, etc. Some short messages are also rewritten into "quizzes", where the educational content is rephrased into a yes or no type question. When answering a quiz, a follow-up answer is displayed to participants stating the correct answer with additional explanation.

3.2.2 The SELFBACK mobile app

The SELFBACK app has been developed as part of the SELFBACK project funded by the European Union Horizon 2020 research and innovation program. The SELFBACK app has been tested in a pilot study and is currently undergoing testing in a larger RCT in people with LBP in primary care. Participants access their weekly self-management plan via the SELFBACK app and enter data into the DSS by answering tailoring questions in the app. These data are combined with participants self-reported outcomes collected at baseline or latest follow-up questionnaire. Participants are required to use the SELFBACK app at least once a week to be offered a new self-management plan for the coming week. The app will send push-notifications reminding participants to open the app and view their new self-management plan. Participants can disable or adjust the frequency of notifications in the app settings. The goal of the intervention is that participants learn to self-manage their LBP and/or NP, which may potentially lead to participants discontinuing the use of the app. Therefore, discontinuation is not necessarily a sign of low

compliance but may indicate higher self-management levels. Nevertheless, to accommodate fluctuations in use of the app a “welcome back” sequence is constructed to guide participants back into the intervention if they re-open the app after more than 4 weeks.

3.3 e-Help webpage in addition to usual care (intervention group 2)

Participants randomized to receive the e-Help webpage will receive an email with a link to access the webpage. The e-Help is an evidence-based resource providing self-management content equivalent to the SELFBACK including educational messages, physical activity advises and exercise recommendations. The exercise material is presented in short videos along with instructions on how to build an exercise program and progression, however no tailoring of the content is offered in this solution. As such, no additional outcome data will be collected from participants randomized to receive the e-Help webpage beyond the regular follow up assessments. Participants will be advised to access the e-Help webpage regularly throughout the study period. Participants randomized to this group may continue to seek care, treatment or help elsewhere as normal.

3.4 Ancillary and post-trial care

Access to the SELFBACK app and the e-Help webpage will cease after the last follow-up by disabling the participants username in the system. No further post-trial care is planned.

4. Outcomes

All outcomes will be collected at baseline, and then six weeks, three months and six months after baseline. Demographic and health-related variables including age, sex, ethnicity, height, weight, educational status, employment, and relevant comorbidities will be collected at baseline. The timeline for data collection is reported in **Table 1** and described below.

Additionally, at the beginning of the study participants will be asked to fill out self-reported Fibromyalgia Survey Criteria [18] and the *Keele StarT MSK* tool which is a prognostic tool for patients with musculoskeletal pain developed to support clinical decisions by stratifying patients into three risk categories (high, medium and low) [19]. The Keele StarT MSK tool includes 10 items assessing psychosocial factors, function and disability, comorbidity and the impact of pain. The total score ranges from 0 to 12 where 0-4 score is classified as low risk, 5-8 as medium risk and 9-12 as high risk [19]. In addition, two items related to return to work will be administered to those in employment to predict short and long terms absence from work.

4.1 Primary outcome

The Musculoskeletal Health Questionnaire (MSK-HQ) measures the multidimensional *impact* of a musculoskeletal (MSK) condition on a person’s health and is chosen as primary outcome. The MSK-HQ is particularly suited for this study as it was designed to be applicable across various MSK conditions and clinical pathways, particularly primary and intermediate care settings [20]. The MSK-HQ contains 14 items assessing key domains of MSK health status relevant to the patients including severity of pain/stiffness, physical function, physical activity level, symptom interference, sleep, fatigue, emotional well-being, understanding diagnosis and treatment, confidence to self-manage, independence and overall impact of symptoms. Each item is scored from 0 to 4 and scores from all items are summed to provide a final score ranging from 0 to 56, with higher scores indicating better MSK health status.

The questionnaire has shown excellent test-retest reliability, high internal consistency [20] and adequate responsiveness to change in four validation cohorts [21]. The minimal clinical important difference for the MSK-HQ has been reported to be 5.5 points (95% CI 2.7 to 8.3) [21]. The questionnaire is translated and available in Norwegian language.

4.2 Secondary outcome

Pain-related disability will be measured by the Roland Morris Disability questionnaire (RMDQ) [22] and by the Neck Disability Index (NDI) [23]. The RMDQ consists of 24 items asking participants to indicate if they experience functional impairments by answering 'yes' or 'no' to a series of descriptions of functional abilities. The NDI consists of 10 items asking participants to rate their functional abilities related to neck pain. Each item is rated on a scale from 0 to 5 and the final score is 50 with higher score indicating higher pain-related disability.

The *average* and *worst LBP/NP intensity* within the past week will be assessed using a 11-point numerical rating scale (NRS) ranging from 0='no pain' to 10='worst pain imaginable'.

The *Pain duration* will be measured by asking the following two questions: 1) "What is the length of time you have had LBP/NP during this episode?", and 2) "What is the total length of time that you have had LBP/NP during the last 12 months?".

Pain medication frequency intake will be measured by asking "How many days during the last week have you taken non-prescription pain medication for LBP/NP?".

The *Fear-Avoidance Belief Questionnaire (FABQ)* assesses participant's beliefs about how physical activity and work affect their LBP and/or NP [24]. The FABQ is a 5-item questionnaire, where participants score their beliefs about their LBP/NP on an ordinal scale ranging from 0='completely disagree' to 6='completely agree'. This scale was slightly modified by changing the word 'back' to 'back or neck' as reported in other studies [25, 26].

The *Pain Self-Efficacy Questionnaire* (PSEQ) assesses participants' level of confidence in carrying out specific activities despite their pain [27]. The PSEQ is a 10-item questionnaire scored on an ordinal scale ranging from 0 ='completely disagree' to 6='completely agree'.

Activity Limitation questionnaire evaluates whether LBP/NP has been limiting for work and leisure activities. This consists of two single items with response options 'yes' and 'no'.

Work Ability is measured by the single-item work ability index (WAI) question and rated on an 11-point NRS scale ranging from 0='completely unable to work' to 10='work ability at its best' [28].

The revised version of *Saltin-Grimby Physical Activity Level Scale* will be used to evaluate self-reported physical activity. Participants are asked to indicate the amount of time per week performing leisure activities with four levels of intensity ranging from sedentary to vigorous physical activity [29].

The *Patient Specific Functional Scale* (PSFS) will be used to evaluate function. Participants are asked to rate their ability to perform up to two self-selected activities regarded as important by them [30]. The ability to carry out the activities is rated from 0='unable to perform' to 10='completely able to perform'.

Sleep problems will be assessed by four self-report items about problems with falling asleep, waking up repeatedly, waking up too early, and feeling sleepy during the day [31]. Items are scored as 'seldom or

never', 'sometimes', and 'several times a week'. Responses to these four items will provide information needed to diagnose insomnia according to the DSM-V criteria [32].

The *Perceived Stress Scale (PSS)* will be used to evaluate stress levels [33]. This consists of 10 items asking about frequency of thoughts and feelings related to perceived stress rated on a 5-point Likert scale ranging from 0='never' to 4='very often'.

The *EuroQoL 5-dimension* (EQ-5D) questionnaire will be used to assess health-related quality of life [34]. This consists of 5 dimensions, i.e. mobility, self-care, activities, pain/discomfort and anxiety/depression rated on a 5-point Likert scale ranging from 'no problem' to 'complete inability'.

The *Brief Illness Perception Questionnaire* (BIPQ) will be used to evaluate participants' illness perception [35]. This consists of 8 items that are scored on an ordinal scale ranging from 0='no problem' to 10='worst severity'.

The *Patient Health Questionnaire-8* (PHQ-8) will be used to evaluate patients' depressive symptoms [36]. This consists of 8 items scored on a 4-point Likert scale ranging from 0='not at all' to 4='nearly every day' assessing the frequency of experiencing symptoms of depression.

Patient Acceptable Symptom State (PASS) will be used to determine which patients consider themselves well and, as such, are satisfied with the treatment [37].

Patient's Global Perceived Effect will be used as a single item question to investigate participants' perception of effect from the intervention they have received [38].

4.3 Other outcomes

Participants randomized to the SELFBACK intervention will be asked a set of weekly tailoring questions to individualize the self-management plans. Participants will be asked a maximum of 7 questions per week (usually 3-4). These include pain intensity (11-point NRS), function (item 5 from the Chronic Pain Grade Questionnaire [39]), fear-avoidance (item 1 Tampa scale [40]), workability (single-item WAI [28]), sleep (single-item PSS [31]), symptoms of depression (2 items from PHQ-8 [36]), and barriers of self-management (single-item, customized to SELFBACK). The selection of questions is based on a set of rules implemented in the backend of the DSS which takes into account the progression of the self-management and the participant's characteristics.

Table 1 Data collection timeline

Baseline	Follow-ups	Weekly (SELFBACK)
Demographic variables	**MKS-HQ***	Pain intensity
**MKS-HQ***	Disability level	Function
Disability level	Pain intensity and duration	Fear avoidance
Pain intensity and duration	Pain medications	Work ability
Pain medications	Pain-related cognitions	Sleep
Pain-related cognitions	Physical activity level	Depressive symptoms
Physical activity level	Function	Barriers to SM
Function	Activity limitation	
Activity limitation	Work ability	
Work ability	Sleep	
Sleep	Mood (stress and depressive symptoms)	

Mood (stress and depressive symptoms)	Health-related quality of life	
Health-related quality of life	Illness perception	
Illness perception	Patients acceptable symptom state	
	Patient's global perceived effect	

* Primary outcome. MSK-HQ: Musculoskeletal Health Questionnaire; SM: self-management

5. Statistics

5.1 Sample size estimation

This RCT is a superiority study with three parallel groups. Participants with LBP and/or NP on a waiting list for rehabilitation will be randomized to receive: 1) the SELFBACK intervention in addition to usual care (intervention group 1); 2) the e-Help webpage in addition to usual care (intervention group 2); or 3) usual care only (control group). We will test the hypothesis that the SELFBACK group will have a 4-point improvement in musculoskeletal health, assessed by MSK-HQ, compared to the e-Help webpage group and the usual care group over the 3 months follow-up period. The minimally important change for MSK-HQ is 5.5 (95% CI 2.7-8.3). This calculation was based on data from 610 individuals with musculoskeletal conditions recruited from four various clinical pathways; patients treated with physiotherapy for a range of musculoskeletal conditions in primary care and patients undergoing hip replacement, knee replacement or shoulder surgery in secondary care (https://innovation.ox.ac.uk/outcome-measures/musculoskeletal-health-questionnaire-msk-hq/).

The sample size calculations have been performed in two ways. First, we conducted a simple calculation assuming only one follow-up measure and a standard deviation (SD) of the MSK-HQ score of 10 points. Based on this calculation we estimated that a sample size of 396 (132 per arm) was necessary to detect a 4-point difference with 90% power and a two-sided alpha level of 0.05. We then performed a simulation using 2000 repetitions of a mixed model regression for repeated measures, assuming 1) three data points per participant (i.e., baseline, 6 weeks, and 3 months), 2) an effect of treatment of 4 points on the MSK-HQ, 3) an SD of 10 points, 4) a correlation between repeated measures of 0.4, and a two-sided alpha level of 0.05. Based on these assumptions, sample size calculations showed that 360 participants (i.e., 120 participants in each group) gave a power of 91% (95% confidence interval [CI 90-92]) to detect a 4-point difference in MSK-HQ score between study groups at 3 months. A recent systematic review showed that attritions rates ranged between 4-94% for digital self-management interventions lasting between two weeks and 12 months in LBP populations [14]. To allow for a 20% drop out rate at 3 months follow-up we aim at including a total of 432 participants in the trial; 144 participants in each arm.

6. Data collection and management

6.1 Data collection

Data will be collected online directly from participants. Participants will be sent an email with a link that directs them to the web-based baseline questionnaire using their username and password provided at the start of the trial. To ensure a high response rate at follow-ups, one reminder e-mail will be sent after one week and an additional e-mail after two weeks. However, for the 6-week assessment a reminder email will be sent after 2 days to ensure timely collection of data before the start of the rehabilitation program. For the primary outcome at three months, a researcher will call the participant if there is no response after 4 days after sending the reminder email. The researcher will then ask if the patient is

willing to answer the primary outcome, i.e. MSK-HQ on the phone. Three attempts will be made at different days of the week and times of day.

In addition to the outcomes obtained at baseline and follow-ups, participants in the intervention group 1 will answer a set of tailoring questions on a weekly basis as described in section 4.3. These will be collected online as described above.

6.2 Data management

6.2.1 Handling personally identifiable data

Upon enrolment into the trial, participants will be assigned an identification number for the study. A key document, linking the identification number to participants' name, email address, and phone number will be created and kept securely at the research facility. The data collection process will be automated in order to minimise the potential for error in the data collection. Email addresses for included participants will be securely stored at the research facility, enabling the system to automatically send emails to the participants with links to the questionnaire website. At the website, participants log in and access the questionnaires at baseline and follow-ups. To safeguard the email addresses, the link between participant identification number and email address is kept in a database separate from the database were outcome data are stored. Consequently, only the id number is registered in the system, whereby all personally identifiable data are kept separate from the DSS and apps.

6.2.2 Data security

All outcome data is stored at a secure server at Department of Computer Science, NTNU, Norway. The servers are firewall protected. The entire virtual machine is backed-up daily, and back-ups are kept for a one-year period. Data storage is compliant with existing European law.

The SELFBACK servers can only be accessed by the technical staff at NTNU's Department of Computer Science (Kerstin Back, Ilya Ashikhmin). Additional access can only be approved by the responsible technical staff (Kerstin Bach). Researchers connected to the recruitment of participants, data collection and conduct of the trial are not allowed to add data or to review, access, or make changes in original participant data. Also, any events in the servers are logged in a log-file to be able to review events or changes to the database. Finally, no information concerning group allocation is held in the outcome database, this information is kept in the WebCRF system (as described in section 2.7), which is separate from the entire SELFBACK system.

7. Data monitoring

7.1 Harms

No serious adverse events are expected for this trial. Regarding the SELFBACK intervention, as the self-management plans may include advice to increase physical activity and exercise volume, increased muscle soreness and transient increase in joint pain are expected. Such symptoms are well known in exercise interventions and as they are transient, they pose no harm to the participants [41]. Additionally, participants are informed that such events may occur and that they are normal. Further, any detection of unusual pain increase is automatically reacted to by the DSS system and a suggestion to adjust volume of physical activity or exercise and advice on handling muscle pain is given to the

participant. In addition, a checklist can be consulted within the app if participants are experiencing worsening of symptoms or pain flare-ups. In the checklist, participants are advised to seek care with their primary care provider or emergency clinics as they normally would. Consequently, as serious adverse events are unexpected no interim analysis or a priori defined stopping rules are defined or implemented for this trial. However, in the unlikely event of an adverse event this will be fully recorded and reported to Norwegian health authorities in line with EU Guidelines on Medical Devices (MEDDEV 2.7/3), Clinical Investigations: Serious adverse event reporting under directives 90/385/eec and 93/42/eec[1].

A telephone hotline will be established where participants can seek technical support for any questions relative to the use of the app during office hours or by leaving a message asking to be contacted the following day. Although the SELFBACK app and the e-Help webpage are designed to be self-explanatory, adequate training on how to use the app and the webpage will be provided via instruction videos and through the possibility to call the hotline. Also, the SELFBACK app will link to a website with a Frequently Asked Questions section that can guide participants with technical issues. Should a participant call the telephone hotline concerning any worsening of symptoms, the participant will be advised to seek care from their health care professional as they normally would if not included in the trial. All enquiries to the telephone hotline will be recorded and discussed in an internal audit and reported with the study results.

We do not foresee any adverse events related to the use of the e-Help webpage. Nevertheless, the telephone hotline described above will also be available for users of the e-Help webpage concerning technical issues.

7.2 Auditing

On a monthly basis, the project leader (Sigmund Gismervik), a secretary at the clinic involved in patient recruitment, the chief physician assessing patient referrals, and a member of the research team will review the recruitment, enrolment, data collection, conduct of the intervention, completion of the trial, reported adverse events and discuss appropriate actions to any inconsistencies or unexpected events. The purpose of this internal audit is to detect any inconsistency between the planned trial conduct and the performed trial conduct as well as suggesting measures to address such inconsistencies. In the unlikely case of serious events that may be related to the conduct of the trial, the principal investigator will be responsible on decisions about premature suspension.

8. Ethics and dissemination

8.1 Research ethics approval

Authorization from the Regional Committee for Medical and Health Research Ethics is to be sought before the commencement of any research-related activity. Approval from institutional review board and/or data protection will be obtained by the National Data Protection Authority and/or the Centre for Research Data.

[1] http://ec.europa.eu/DocsRoom/documents/16477/attachments/1/translations/en/renditions/pdf

8.2 Protocol amendments

Any amendments to the protocol will be registered with a detailed description of the change and date of implementation. Any amendments to the protocol will be filed with the relevant ethical committees or data protection agencies and registered in the clinical trial registry (www.clinicaltrials.gov) for transparency. Any amendments to the protocol related to the SELFBACK app will be submitted to the Norwegian Medicines Agency for approval.

8.3 Declaration of interests

The aim of this project is to test the effectiveness of the SELFBACK app and the e-Help website in improving musculoskeletal health among patients on waiting list for multidisciplinary treatment for LBP and/or NP at an outpatient clinic. The results and experiences from the RCT may inform the further development of the app. The SELFBACK app may be introduced into a commercial market. In order to secure an unbiased interpretation and dissemination of the RCT, the interpretation of the results will therefore be performed blind to group allocation. Upon publication of study results, this commercial potential of the SELFBACK app will be clearly stated and the publication will undergo peer-review to ensure methodological and scientific rigor.

8.4 Access to data

NTNU will have sole ownership of the data collected. All personal identifiable data collected in the trial will be kept for five years. Hereafter the data set will be fully anonymised. These data are kept to enable tracking of any adverse events reported post completion of the trial, and to enable the project to contact enrolled participants should any plan of additional long-term follow-ups be necessary. The anonymised full data set will be kept for 30 years for research purposes.

8.5 Dissemination policy

The results of this RCT will be disseminated through publications in peer-reviewed journals as well as through reports and presentations at national and international conferences relevant to this research topic.

References

1. Chan, A.-W., et al., *SPIRIT 2013 statement: defining standard protocol items for clinical trials.* Annals of internal medicine, 2013. 158(3): p. 200-207.
2. Schulz, K.F., D.G. Altman, and D. Moher, *CONSORT 2010 statement: updated guidelines for reporting parallel group randomised trials.* BMC medicine, 2010. 8(1): p. 18.
3. Hagen, K., et al., *Increasing Prevalence of Chronic Musculoskeletal Complaints. A Large 11-Year Follow-Up in the General Population (HUNT 2 and 3).* Pain Medicine, 2011. 12(11): p. 1657-1666.
4. Picavet, H. and J. Schouten, *Musculoskeletal pain in the Netherlands: prevalences, consequences and risk groups, the DMC3-study.* Pain, 2003. 102(1-2): p. 167-178.
5. Vos, T., et al., *Global, regional, and national incidence, prevalence, and years lived with disability for 310 diseases and injuries, 1990–2015: a systematic analysis for the Global Burden of Disease Study 2015.* The Lancet, 2016. 388(10053): p. 1545-1602.
6. Gore, M., et al., *The burden of chronic low back pain: clinical comorbidities, treatment patterns, and health care costs in usual care settings.* Spine, 2012. 37(11): p. E668-E677.
7. Manchikanti, L., et al., *Comprehensive review of epidemiology, scope, and impact of spinal pain.* Pain physician, 2009. 12(4): p. E35-70.
8. Hoy, D., et al., *The global burden of neck pain: estimates from the global burden of disease 2010 study.* Annals of the rheumatic diseases, 2014. 73(7): p. 1309-1315.
9. Hartvigsen, J., et al., *What low back pain is and why we need to pay attention.* The Lancet, 2018. 391(10137): p. 2356-2367.
10. Buchbinder, R., et al., *Low back pain: a call for action.* The Lancet, 2018. 391(10137): p. 2384-2388.
11. Hayden, J.A., M.W. Van Tulder, and G. Tomlinson, *Systematic review: strategies for using exercise therapy to improve outcomes in chronic low back pain.* Annals of internal medicine, 2005. 142(9): p. 776-785.
12. O'Connor, S., et al., *Understanding factors affecting patient and public engagement and recruitment to digital health interventions: a systematic review of qualitative studies.* BMC medical informatics and decision making, 2016. 16(1): p. 120.
13. Machado, G.C., et al., *Smartphone apps for the self-management of low back pain: A systematic review.* Best Practice & Research Clinical Rheumatology, 2016. 30(6): p. 1098-1109.
14. Nicholl, B.I., et al., *Digital Support Interventions for the Self-Management of Low Back Pain: A Systematic Review.* J Med Internet Res, 2017. 19(5): p. e179.
15. Mork, P.J. and K. Bach, *A decision support system to enhance self-Management of low Back Pain: protocol for the selfBACK project.* JMIR research protocols, 2018. 7(7): p. e167.
16. Aamodt, A. and E. Plaza, *Case-based reasoning: Foundational issues, methodological variations, and system approaches.* AI communications, 1994. 7(1): p. 39-59.
17. Montani, S. and L.C. Jain, *Successful case-based reasoning applications*. Vol. 305. 2010: Springer.
18. Wolfe, F., et al. *2016 Revisions to the 2010/2011 fibromyalgia diagnostic criteria*. in *Seminars in arthritis and rheumatism*. 2016. Elsevier.
19. Campbell, P., et al., *Keele Aches and Pains Study protocol: validity, acceptability, and feasibility of the Keele STarT MSK tool for subgrouping musculoskeletal patients in primary care.* Journal of pain research, 2016. 9: p. 807.
20. Hill, J.C., et al., *Development and initial cohort validation of the Arthritis Research UK Musculoskeletal Health Questionnaire (MSK-HQ) for use across musculoskeletal care pathways.* BMJ open, 2016. 6(8): p. e012331.

21. Price, A.J., et al., *Determining responsiveness and meaningful changes for the Musculoskeletal Health Questionnaire (MSK-HQ) for use across musculoskeletal care pathways.* BMJ open, 2019. 9(10): p. e025357.
22. Roland, M. and R. Morris, *A Study of the Natural History of Back Pain: Part IDevelopment of a Reliable and Sensitive Measure of Disability in Low-Back Pain.* spine, 1983. 8(2): p. 141-144.
23. Vernon, H. and S. Mior, *The Neck Disability Index: a study of reliability and validity.* Journal of manipulative and physiological therapeutics, 1991.
24. Waddell, G., et al., *A Fear-Avoidance Beliefs Questionnaire (FABQ) and the role of fear-avoidance beliefs in chronic low back pain and disability.* Pain, 1993. 52(2): p. 157-168.
25. Landers, M.R., et al., *The use of fear-avoidance beliefs and nonorganic signs in predicting prolonged disability in patients with neck pain.* Manual therapy, 2008. 13(3): p. 239-248.
26. Cleland, J.A., J.M. Fritz, and J.D. Childs, *Psychometric Properties of the Fear-Avoidance Beliefs Questionnaire and Tampa Scale of Kinesiophobia in Patients with Neck Pain.* American Journal of Physical Medicine & Rehabilitation, 2008. 87(2): p. 109-117.
27. Nicholas, M., *Pain self efficacy questionnaire (PSEQ).* Summary of psychometric properties from original Westmead hospital sample (1986e87). Sydney: University of Sydney Pain Management and Research Centre, 1994.
28. Ahlstrom, L., et al., *The work ability index and single-item question: associations with sick leave, symptoms, and health–a prospective study of women on long-term sick leave.* Scandinavian journal of work, environment & health, 2010: p. 404-412.
29. Grimby, G., et al., *The "Saltin–Grimby Physical Activity Level Scale" and its application to health research.* Scandinavian journal of medicine & science in sports, 2015. 25: p. 119-125.
30. Hefford, C., et al., *The patient-specific functional scale: validity, reliability, and responsiveness in patients with upper extremity musculoskeletal problems.* journal of orthopaedic & sports physical therapy, 2012. 42(2): p. 56-65.
31. Engstrøm, M., et al., *The reliability of a new sleep screening questionnaire for large population-based studies: the third nord-trøndelag health study.* The Open Sleep Journal, 2011. 4(1).
32. Association, A.P., *Diagnostic and statistical manual of mental disorders (DSM-5®).* 2013: American Psychiatric Pub.
33. Cohen, S., T. Kamarck, and R. Mermelstein, *A global measure of perceived stress.* Journal of health and social behavior, 1983: p. 385-396.
34. Brooks, R. and E. Group, *EuroQol: the current state of play.* Health policy, 1996. 37(1): p. 53-72.
35. Hallegraeff, J.M., et al., *Measurement of acute nonspecific low back pain perception in primary care physical therapy: reliability and validity of the brief illness perception questionnaire.* BMC musculoskeletal disorders, 2013. 14(1): p. 53.
36. Kroenke, K., et al., *The PHQ-8 as a measure of current depression in the general population.* Journal of affective disorders, 2009. 114(1-3): p. 163-173.
37. Kvien, T.K., T. Heiberg, and K.B. Hagen, *Minimal clinically important improvement/difference (MCII/MCID) and patient acceptable symptom state (PASS): what do these concepts mean?* Annals of the rheumatic diseases, 2007. 66(suppl 3): p. iii40-iii41.
38. Kamper, S.J., et al., *Global Perceived Effect scales provided reliable assessments of health transition in people with musculoskeletal disorders, but ratings are strongly influenced by current status.* Journal of clinical epidemiology, 2010. 63(7): p. 760-766. e1.
39. Von Korff, M., et al., *Grading the severity of chronic pain.* Pain, 1992. 50(2): p. 133-149.
40. Verwoerd, A.J., et al., *A single question was as predictive of outcome as the Tampa Scale for Kinesiophobia in people with sciatica: an observational study.* Journal of physiotherapy, 2012. 58(4): p. 249-254.

41. Olsen, O., et al., *The effect of warm-up and cool-down exercise on delayed onset muscle soreness in the quadriceps muscle: a randomized controlled trial.* Journal of human kinetics, 2012. 35(1): p. 59-68.

An individually tailored self-management app-based intervention (SELFBACK) versus a self-management web-based intervention (e-Help) or usual care in people with low back and neck pain referred to secondary care: protocol for a multi-arm randomised clinical trial

Section 1: Administrative Information

SAP Version 1.0 (03.08.2021)
DOI: 10.5281/zenodo.5155811

This SAP is based on the protocol registered in ClinicalTrials.gov (NCT04463043) and the submitted protocol paper. The structure and content of the SAP is adopted from the Guidelines for the Content of Statistical Analyses Plans in Clinical Trials [1].

Table 1. SAP revision history

Revision	Justification for revision	Version (date)

Signatures

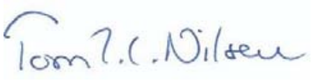

Senior Statistician

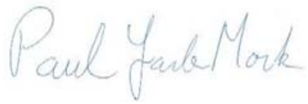

Project Coordinator

Section 2: Introduction

2.1 Background and rationale

Self-management is a key element in the care of low back pain (LBP) and neck pain (NP). Current best evidence recommends that self-management is tailored to individual needs and capabilities and includes elements such as education, exercise programs, and advice to stay active[2]. In primary care, general practitioners commonly lack time, resources and training for delivering evidence-based self-management support[3], while access to specialist care for patients with more complex symptoms is generally limited and requires long waiting time. Digital solutions, such as mobile applications (apps) provide a viable option for supporting tailored self-management across care pathways as they can be accessible to patients at any time and at low cost. The role of digital interventions as an adjunct to secondary care have not yet been explored. Further, the added benefit of a tailored over a non-tailored approach is currently unclear.

We have developed an evidence-based and data-driven decision support system (DSS) delivered via a smartphone app – SELFBACK – to facilitate, improve and reinforce individually tailored self-management of non-specific LBP, which has been adapted to also target NP[4]. Additionally, we have developed a website – eHelp – that mimics the content of the SELFBACK app, but without individual tailoring. The effectiveness of these digital interventions is evaluated in a secondary care setting in a multi-arm randomised clinical trial (RCT).

2.2 Objectives

The primary objective is to evaluate the effectiveness of the SELFBACK DSS as adjunct to usual care versus usual care only. The secondary objective is to compare the effectiveness of the SELFBACK DSS to the e-Help website as well as e-Help vs usual care. The primary outcome is musculoskeletal health at three months measured by the Musculoskeletal Pain Questionnaire (MSK-HQ).

The effect of the interventions on secondary outcomes, including quality of life, use of non-prescriptive medication, sleep problems, depressive symptoms, stress, functional ability, and pain intensity, will be assessed at three months. We will also evaluate the effect on these measures, as well as on MSK-HQ, at six months.

Section 3: Study Methods

3.1 Trial design

This study is designed as a multi-arm RCT where patients with LBP and/or NP are randomised to three parallel groups (allocation ratio 1:1:1). The intervention groups will be given access to the SELFBACK DSS delivered via a smartphone app, or to the e-Help website, additional to usual care, whereas the control group will get usual care only. The target population is people referred to secondary care for LBP and/or NP at the multidisciplinary clinic for back-, neck-, and shoulder rehabilitation at St. Olavs Hospital, Trondheim, Norway; see also sections 5.2 and 5.3.

3.2 Randomization

Randomisation is performed as a permuted block randomisation of random size unknown to the research team to ensure allocation concealment. Randomisation is performed by a web-based randomisation system (Web Case Report Form; WebCRF) developed and administered by Unit of Applied Clinical Research, Faculty of Medicine and Health Sciences, NTNU, Trondheim, Norway. This unit is not otherwise involved in the trial management or trial conduct.

3.3 Sample size

The sample size calculation is described in detail in the registered protocol. Briefly, the study aims to detect a four-point difference between the intervention groups, i.e. SELFBACK and e-Help and the control group in musculoskeletal health measured by the MSK-HQ at three months follow-up. Using the *sampsi* procedure within Stata, assuming a standard deviation of the MSK-HQ score of 10 points and a correlation between repeated measures of 0.4 and a 30% drop-out, a sample size of at least 279 participants (93 in each arm) will be included in this trial.

3.4 Framework

This trial is designed as a superiority RCT assessing the effectiveness of digital interventions (i.e., the SELFBACK DSS or the e-Help website) in addition to usual care, compared to usual care only (control group) for people with LBP and/or NP. Three comparisons will be performed, SELFBACK vs usual care (primary analysis), e-Help vs usual care and SELFBACK vs e-Help (secondary analyses).

3.5 Interim analyses and stopping guidance

As serious adverse events are unexpected, no interim analysis or a priori defined stopping rules are defined or implemented for this trial.

3.6 Timing of outcome assessment

The primary and secondary outcome variables will be assessed at baseline and at six weeks, three months, and six months follow-up. This allows analyses of repeated measures on both primary and secondary outcomes, and thus increased statistical power compared to outcomes assessed at a single time-point.

3.7 Timing of final analyses

The analyses of the primary outcome will be conducted within three months after the last participant has completed the six months follow-up questionnaire. The inclusion of participants continued until April 2021, and thus the final analyses should be ready by end of January 2022. Analyses of secondary outcomes assessed at three and six months will be analysed subsequently to the primary outcome and conducted within the same time frame.

Section 4: Statistical Principles

4.1 Confidence intervals and P-values

As recently recommended in the medical literature, we will not use a specific *P*-value threshold to decide upon statistical significance as this often leads to misinterpretation of results. For the same reasons, we will not adjust for multiple comparisons since this build upon a strict use of a certain *P*-value threshold. Instead, the precision of the estimated effects of the intervention will be assessed by a 95% confidence interval, and the effect will be described as a point estimate (mean difference or odds ratio/relative risk) with accompanying confidence limits. Whenever *P*-values are reported, we will do so by presenting their actual value, and not reduce them to a binary inequality under or above a threshold value.

4.2 Adherence and protocol deviations

The first login to the SELFBACK app and e-Help website will be monitored to check if participants accessed the allocated intervention. Participants who do not access the intervention after the reminders or want to discontinue its use will be followed up as usual. Data analytics on usage and interaction with the app/web interventions will be available as an indirect measure of adherence. There is currently no optimal way to measure adherence in self-management interventions [5-7] which makes it challenging to establish a per-protocol analysis. Additionally, there is a risk that a per-protocol analysis can be biased if participants who engage with the digital intervention have different prognosis from those who engage less with it. As such, the primary analysis will include all participants enrolled in the study (see 4.3 for details), but we will conduct supplementary exploratory analyses in subgroups according to different definitions of adherence: 1) restricted to participants who have accessed the app or web page; and 2) have had a certain level of engagement with the SELFBACK app, e.g. having generated six self-management plans during the first twelve weeks after enrolment.

4.3 Analysis populations

The main effect of the intervention will be analysed according to the intention-to-treat principle using linear mixed models for continuous outcomes and generalized estimated equations (GEE) for binary outcomes (logistic and Poisson models), and the analyses will include all participants initially enrolled in the study and who answered the baseline questionnaire and were randomised. The web-based baseline questionnaire does not allow participants to proceed without filling in an answer, so there will be no missing data at this time point. A similar solution will be used for the follow-up questionnaires, but missing data will be generated if participants do not answer the follow-up questionnaire (i.e. due to withdrawal or loss to follow-up).

Any missing values throughout the follow-up period are inherently accounted for in the mixed model approach and complete case analysis will be applied in sensitivity analyses (see chapter 6.2 and 6.3 below for further details). A complete case will be defined as a participant who has answered both the baseline and the three-month questionnaire. We will also conduct supplementary exploratory analyses only including participants from the intervention arm who are defined as adhering to the intervention (see chapter 4.2 for more details).

Section 5: Trial Population

5.1 Screening data

The trial does not aim to collect any screening data to describe the representativeness of the sample.

5.2 Eligibility

Detailed inclusion and exclusion criteria are described in the registered protocol. Briefly, participants must be ≥18 years, referred and accepted to a secondary care hospital outpatient clinic for LBP and/or NP and own a smartphone with internet access. Participants are excluded if they have 'red flags' indicating possible serious pathology, if they are unable to take part in exercise of physical activity or unable to speak and/or read Norwegian.

5.3 Recruitment

The recruitment of participants is conducted in Trondheim at the multidisciplinary outpatient clinic for back-, neck-, and shoulder rehabilitation at St. Olavs Hospital. The recruitment was conducted between July 2020 and April 2021 and details about the number of participants contacted and included in the trial will be visualised in the CONSORT flow-chart.

5.4 Withdrawal and follow-up

Each participant is informed that they can withdraw from the study at any time, and that they then have the right to have any personal, health and questionnaire data deleted. If a participant withdraws during the follow-up period, but do not require already collected data to be deleted, the data will be used in the analyses until the time point for withdrawal. For analyses of the primary and secondary outcomes at three months, loss to follow-up is defined as not answering the three-month questionnaire. Loss-to-follow-up will be assessed for each outcome variable separately. The same principle to define loss-to-follow-up will be used for the six-month follow-up time point. The number of participants providing information at each follow-up time point will be visualised in the CONSORT flow-chart, and this also displays the number who withdrew or were lost to follow-up between each follow-up time-point.

5.5. Baseline patient characteristics

Eligible participants fill in a baseline web questionnaire after consenting to take part in the study. Baseline characteristics that are collected include: age, sex, height, weight, housing (live alone or with family), education, employment, work characteristics, physical activity, sleep problems, mental health, stress, quality of life, and various pain-related factors (e.g., localisation, duration, intensity, coping, disability and limitations, perceptions and beliefs). Depending on the nature of the variables, we will summarise this information in a baseline table showing mean values with standard deviation or numbers and percentages within the three trial arms. We will not conduct any statistical tests of baseline differences, as this violates the assumptions for the randomisation procedure.

Section 6: Analyses

6.1 Outcome definitions

All outcome variables described below are assessed at baseline, six weeks, three, and six months. The primary follow-up time point is three months, both for the primary and secondary outcome variables described below. For each follow-up period, all measures will inform the analyses (e.g. baseline, six-week and six-month data will be included when analysing effects at three months).

Primary outcome variable

The primary outcome is the mean difference in musculoskeletal health assessed at three months from baseline, measured by the MSK-HQ[8]. The questionnaire contains 14 items assessing severity of pain/stiffness, physical function, physical activity level, symptoms interference, sleep, fatigue, emotional health, understanding of the condition, confidence to self-manage, independence and overall impact of symptoms. Each item is scored from 0 to 4 and are summed to provide a final score (range 0-56), with higher scores indicating better musculoskeletal health. The main analyses will be based on the raw scores, and we will estimate mean group differences in MSK-HQ at three months using a linear mixed model for repeated measures. We will also construct a binary variable representing a clinically meaningful change in MSK-HQ of four points or more during the three months follow-up period that will be analysed using a Poisson GEE analyses for repeated measures to estimate relative risks.

Secondary outcome variables

- *The Roland Morris Disability Questonnaire (RMDQ)* will be used to assess pain-related disability[9]. The RMDQ includes 24 items asking participants to indicate if they experience functional impairments by answering “yes” or “no” to a series of descriptions of functional abilities. The RMDQ score ranges from 0 to 24, where a higher score indicates higher levels of pain-related disability. We will compare mean group differences in RMDQ using a linear mixed model for repeated measures. There is no consensus on what constitute a clinically meaningful improvement on RMDQ, i.e., this may vary from two to four points[10-13]. We will construct a binary variable representing a clinically meaningful change in RMDQ defined as four points.
- *Neck Disability Index (NDI)* will be used to assess neck-specific disability[14]. The questionnaire has 10 items regarding pain and activities of daily living including personal care, lifting, reading, headaches, concentration, work status, driving, sleeping and recreation. The NDI score ranges from 0 to 50 with higher scores indicating greater disability. We will compare mean group differences in NDI using a linear mixed model for repeated measures. Although it is not clear what constitutes a clinically meaningful improvement on NDI[14 15] we will construct a binary variable using 7.5 points as cut-off value.
- *Pain intensity* over the past week will be assessed by asking “Please indicate your average/worst low back and/or neck pain level during the last week“, using an 11-point numerical rating scale (NRS) ranging from “0 (zero)” to “10”[16]. We will compare mean group differences in pain intensity using linear mixed models. We will also construct a binary variable to indicate moderate/severe pain (>5 points).
- *Health-related quality of life* is evaluated with the EuroQoL 5-dimension (EQ-5D) questionnaire[17]. A 5-point Likert scale ranging from “1 [no problems]” to “5 [complete inability]” is used to assess the health-related quality of life within each of

the five dimensions (i.e., mobility, self-care, activities, pain/discomfort and anxiety/depression). We will construct an overall weighted index based on a value set that combines all items and then estimate the mean difference between groups using linear mixed models.

- *General health* is assessed on a 100-point vertical scale where 0 indicates the worst health you can imagine and 100 the best imaginable health[17]. The variable will be analysed as a continuous variable estimating the mean difference between groups using linear mixed models.
- *The Pain Self-Efficacy Questionnaire* (PSEQ) assesses the participant's level of confidence in carrying out specific activities despite their pain[18]. The PSEQ is a 10-item questionnaire scored on an ordinal scale ranging from "zero [completely disagree]" to "six [completely agree]". A total score is calculated by summing the scores for each of the 10 items, yielding a maximum total score of 60, where higher scores reflect stronger self-efficacy beliefs. We will compare mean group differences in PSEQ using linear mixed models. We will also construct a binary variable to indicate low and high self-efficacy using a cut-off value of 40.
- *The Brief Illness Perception Questionnaire* (BIPQ) evaluates the participants' illness perception in an 8-item questionnaire[19]. Items are scored on an ordinal scale ranging from "0 [no problems]" to "10 [worst severity]". Adding the separate score values creates a summary score with a higher score indicating more threatening view of the pain. The summed score will be analysed as a continuous variable to compare mean group differences, and we will also construct a binary variable with cut-offs indicated from the distribution of the variable (e.g. percentiles) since no clinical cut offs are suggested in the literature.
- *The Fear-Avoidance Belief Questionnaire* (FABQ) assesses participant's beliefs about how physical activity and work affect their pain[20]. The FABQ is a 5-item questionnaire, where the participants score their beliefs about their pain on an ordinal scale ranging from "zero [completely disagree]" to "six [completely agree]". The four latter questions will be summed (range 0-24) to represent fear avoidance beliefs about physical activity and analysed as a continuous variable to compare mean group differences using linear mixed models. We will also classify people as having high or low fear for physical activity to examine possible differences in a binary variable. The classification cut-off will be obtained from the distribution of the variable (i.e., median value).
- *Stress* is evaluated with the Perceived Stress Scale (PSS), a 10-item questionnaire asking about frequency of thoughts and feelings related to perceived stress[21]. Participants indicate their frequency of experiencing stress-related issues on a 5-point Likert scale, ranging from "0 [never]" to "4 [very often]". Positive score items are reversed and then all items are summed to a score ranging from 0 to 40. The resulting sum score will be analysed as a continuous variable to estimate mean differences in stress using linear mixed models. A score ≥27 is considered high stress and will be used as cut-off value to construct a binary variable.
- *The Patient Health Questionnaire-8* (PHQ-8) is an 8-item questionnaire used to evaluate the participants' depressive symptoms[22]. Items are reported on a 4-point Likert scale scoring frequency of experiencing symptoms of depression. The nine items will be summed and analysed both as a continuous variable using linear mixed models and as a binary variable using a cut-off 15 to classify people into none/mild/moderate versus moderately severe/severe depression.
- *Function* is evaluated by the Patient Specific Functional Scale (PSFS) where participants are asked to rate up to two self-selected activities they are unable to do or

having difficulties performing[23]. The ability to carry out the activity/activities is rated from “zero [unable to perform]” to “10 [fully able to perform]”. We will compare mean difference in function using linear mixed models.

- *Self-reported physical activity* is evaluated by the Modernised Saltin-Grimby Physical Activity Level Scale, where participants indicate their amount of time per week performing leisure activities with four levels of intensity ranging from sedentary to vigorous physically active[24]. The resulting four categories will be analysed as a binary variable indicating no/light activity vs moderate/vigorous activity.
- *Sleep problems* is assessed by four items including problems with falling asleep, waking up repeatedly, waking up too early, and feeling sleepy during the day[25]. Response options for each item are “seldom or never”, “sometimes” or “several times a week”. The information retrieved from these four items approximates the information necessary to diagnose insomnia according to the DSM-V criteria, and will be analysed as a binary variable (insomnia vs no insomnia).
- *Work ability* is measured by a single-item on current work ability rated on an 11-point NRS scale ranging from “zero [completely unable to work]” to “10 [work ability at its best]”[26]. We will compare mean difference in work ability using linear mixed models. We will also classify people into a binary variable representing high (>7 points) vs low work ability.
- *Patient Acceptable Symptom State* is a single item question: “Considering your pain, do you consider your current state satisfactory?” with response options yes or no[27]. This will be analysed as a binary variable.
- *Patient’s Global Perceived Effect* is a single item question where participants are asked to rate improvement or deterioration of their pain status compared to before the intervention with seven response options ranging from -5 [markedly worse] to 5 [markedly better][28]. The variable will be analysed as a binary variable indicating improved vs not improved.
- *Pain medication* is collected by the question “How many days during the last week have you taken non-prescription pain medication for your pain?” with four response options ranging from “never” to “daily”. Although this information is collected at follow-up the variable will not be included as a secondary outcome.
- *Long term pain duration* is measured by “What is the total length of time that you have had low back or neck trouble during the last 12 months?” with five response options ranging from “0 days” to “every day”. Although this information is collected at follow-up the variable will not be included as a secondary outcome.

6.2 Analyses methods

The primary analysis will estimate mean difference and 95% confidence interval (CI) in MSK-HQ score at three months follow-up between the intervention and control group (i.e., SELFBACK in addition to usual care versus usual care only). The analyses will be conducted according to the intention-to-treat principle using a linear mixed model for repeated measures. This model includes all available data for all participants at each time point (i.e. baseline, six weeks, and three months). The distribution of the MSK-HQ score will be assessed, and the variable may be transformed (e.g. log transformation) to better fit with the assumptions for the regression analyses. In the regression model, individual participants will be specified as a random effect, accounting for the within subject covariance structure. The effect of group and time will be specified as fixed effects using a joint variable of

intervention and time in a constrained longitudinal data analysis approach. Here, baseline levels are pooled over the two study groups assuming that any baseline differences are due to chance; this also controls for any baseline differences in the outcome variable. The same approach will be used for the other two comparisons, i.e. e-Help website vs usual care and SELFBACK vs e-Help website.

All analyses will include adjustment for baseline levels of potentially important prognostic factors, such as age, sex, socioeconomic status, and pain intensity. We will also use Poisson GEE analyses to estimate relative risk (with 95% CI) for a four-point change in MSK-HQ between the groups taking into account the repeated observations. This analysis will be adjusted for the same factors as those included in the linear mixed model.

To reduce the risk of biased interpretation of results for the primary outcome we will draft separate interpretations based on the results from the main analyses, with groups arbitrarily labelled as A, B, and C (Table 2). After agreeing on the interpretations, the randomisation code is broken, and the correct interpretation will be used.

Table 2. An interpretation will be drafted for each of the six possible combinations of group labelling before unblinding the group allocation.

Label	Interpret. 1	Interpret. 2	Interpret. 3	Interpret. 4	Interpret. 5	Interpret. 6
A	SELFBACK	SELFBACK	e-Help	e-Help	Control	Control
B	e-Help	Control	SELFBACK	Control	e-Help	SELFBACK
C	Control	e-Help	Control	SELFBACK	SELFBACK	e-Help

In addition to the intention to treat analyses, we will conduct supplementary exploratory per protocol analyses using information on adherence to the trial as described in chapter 4.2 above.

All secondary outcomes will be analysed using the same approach as described for the primary outcome; linear mixed models will be used to estimate mean differences between groups in continuous variables, and for binary variables we will use Poisson GEE analyses to estimate relative risk. Pre-specified cut-offs for binary variables are described in 6.1 above. For analyses of mean differences, the distribution of each outcome variable will be assessed to inform possible transformation or initiate alternative analytical procedures (e.g. non-parametric analyses). The precision of all estimated effects will be assessed by a 95% CI.

Possible modifiers of the effect of intervention on the primary outcome will be assessed in supplementary analyses stratified by sex, age groups, socioeconomic status and different levels of pain severity etc., and accompanied by tests of statistical interaction to assess departure from additive effects (i.e., including a product term of group and modifier in the regression model).

6.3 Missing data

Any missing values are inherently accounted for in the mixed model/GEE approach assuming that the data are missing at random. We will also conduct complete case analyses including people with data on all time-points.

6.4 Additional analyses

Additional analyses include exploratory analyses, analyses of secondary outcomes, analyses stratified by possible effect modifiers, analyses using multiple imputation of missing data and complete case analyses. These analyses are described above in chapter 6.1-6.3.

6.5 Harms

Since no harms are expected, we do not plan any specific analyses for this. If any study related harms should occur, these will be described and reported.

6.6 Statistical software

All analyses related to the primary outcome will be conducted using Stata.

References

1. Gamble C, Krishan A, Stocken D, et al. Guidelines for the content of statistical analysis plans in clinical trials. *Jama* 2017;318(23):2337-43.
2. Lin I, Wiles L, Waller R, et al. What does best practice care for musculoskeletal pain look like? Eleven consistent recommendations from high-quality clinical practice guidelines: systematic review. *British journal of sports medicine* 2020;54(2):79-86.
3. Kamper SJ, Logan G, Copsey B, et al. What is usual care for low back pain? A systematic review of health care provided to patients with low back pain in family practice and emergency departments. *Pain* 2020;161(4):694-702.
4. Mork PJ, Bach K. A decision support system to enhance self-Management of low Back Pain: protocol for the selfBACK project. *JMIR research protocols* 2018;7(7):e167.
5. Widmer RJ, Senecal C, Allison TG, et al. Dose-Response Effect of a Digital Health Intervention During Cardiac Rehabilitation: Subanalysis of Randomized Controlled Trial. *Journal of medical Internet research* 2020;22(2):e13055.
6. Donkin L, Hickie IB, Christensen H, et al. Rethinking the dose-response relationship between usage and outcome in an online intervention for depression: randomized controlled trial. *Journal of medical Internet research* 2013;15(10):e231.
7. Mattila E, Lappalainen R, Välkkynen P, et al. Usage and dose response of a mobile acceptance and commitment therapy app: secondary analysis of the intervention arm of a randomized controlled trial. *JMIR mHealth and uHealth* 2016;4(3):e5241.
8. Hill JC, Kang S, Benedetto E, et al. Development and initial cohort validation of the Arthritis Research UK Musculoskeletal Health Questionnaire (MSK-HQ) for use across musculoskeletal care pathways. *BMJ open* 2016;6(8):e012331.
9. Roland M, Morris R. A Study of the Natural History of Back Pain: Part IDevelopment of a Reliable and Sensitive Measure of Disability in Low-Back Pain. *spine* 1983;8(2):141-44.
10. Jordan K, Dunn KM, Lewis M, et al. A minimal clinically important difference was derived for the Roland-Morris Disability Questionnaire for low back pain. *Journal of clinical epidemiology* 2006;59(1):45-52.
11. Ostelo RW, Deyo RA, Stratford P, et al. Interpreting change scores for pain and functional status in low back pain: towards international consensus regarding minimal important change. *Spine* 2008;33(1):90-94.
12. Stratford PW, Binkley JM, Riddle DL, et al. Sensitivity to change of the Roland-Morris back pain questionnaire: part 1. *Physical therapy* 1998;78(11):1186-96.
13. Ostelo RW, de Vet HC. Clinically important outcomes in low back pain. *Best Practice & Research Clinical Rheumatology* 2005;19(4):593-607.
14. Pool JJ, Ostelo RW, Hoving JL, et al. Minimal clinically important change of the Neck Disability Index and the Numerical Rating Scale for patients with neck pain. *Spine* 2007;32(26):3047-51.
15. Young BA, Walker MJ, Strunce JB, et al. Responsiveness of the Neck Disability Index in patients with mechanical neck disorders. *The Spine Journal* 2009;9(10):802-08.
16. Hawker GA, Mian S, Kendzerska T, et al. Measures of adult pain: Visual analog scale for pain (vas pain), numeric rating scale for pain (nrs pain), mcgill pain questionnaire (mpq), short-form mcgill pain questionnaire (sf-mpq), chronic pain grade scale (cpgs), short form-36 bodily pain scale (sf-36 bps), and measure of intermittent and constant osteoarthritis pain (icoap). *Arthritis care & research* 2011;63(S11):S240-S52.
17. Brooks R, Group E. EuroQol: the current state of play. *Health policy* 1996;37(1):53-72.
18. Nicholas MK. The pain self-efficacy questionnaire: taking pain into account. *European journal of pain* 2007;11(2):153-63.
19. Hallegraeff JM, van der Schans CP, Krijnen WP, et al. Measurement of acute nonspecific low back pain perception in primary care physical therapy: reliability and validity of the brief illness perception questionnaire. *BMC musculoskeletal disorders* 2013;14(1):1-7.

20. Waddell G, Newton M, Henderson I, et al. A Fear-Avoidance Beliefs Questionnaire (FABQ) and the role of fear-avoidance beliefs in chronic low back pain and disability. *Pain* 1993;52(2):157-68.
21. Cohen S, Kamarck T, Mermelstein R. A global measure of perceived stress. *Journal of health and social behavior* 1983:385-96.
22. Kroenke K, Strine TW, Spitzer RL, et al. The PHQ-8 as a measure of current depression in the general population. *Journal of affective disorders* 2009;114(1-3):163-73.
23. Hefford C, Abbott JH, Arnold R, et al. The patient-specific functional scale: validity, reliability, and responsiveness in patients with upper extremity musculoskeletal problems. *journal of orthopaedic & sports physical therapy* 2012;42(2):56-65.
24. Grimby G, Börjesson M, Jonsdottir I, et al. The "Saltin–Grimby Physical Activity Level Scale" and its application to health research. *Scandinavian journal of medicine & science in sports* 2015;25:119-25.
25. Engstrøm M, Ødegård SS, Sand T, et al. The reliability of a new sleep screening questionnaire for large population-based studies: the third nord-trøndelag health study. *The Open Sleep Journal* 2011;4(1)
26. Ahlstrom L, Grimby-Ekman A, Hagberg M, et al. The work ability index and single-item question: associations with sick leave, symptoms, and health–a prospective study of women on long-term sick leave. *Scandinavian journal of work, environment & health* 2010:404-12.
27. Salaffi F, Carotti M, Gutierrez M, et al. Patient acceptable symptom state in self-report questionnaires and composite clinical disease index for assessing rheumatoid arthritis activity: identification of cut-off points for routine care. *BioMed research international* 2015;2015
28. Kamper SJ, Ostelo RW, Knol DL, et al. Global Perceived Effect scales provided reliable assessments of health transition in people with musculoskeletal disorders, but ratings are strongly influenced by current status. *Journal of clinical epidemiology* 2010;63(7):760-66. e1.
