# Supplementary material for: Effect of an Artificial Intelligence–Based Self-Management App on Musculoskeletal Health in Patients With Neck and/or Low Back Pain Referred to Specialist Care: A Randomized Clinical Trial
Source: JAMA Netw Open. 2023 Jun 27;6(6):e2320400. doi: 10.1001/jamanetworkopen.2023.20400 (PMC10300712; doi:10.1001/jamanetworkopen.2023.20400)
Supplement: Supplement 2. — eFigure. Start Screen of the SELFBACK App (Left) and 3 Main Components of the Weekly Self-Management Plan (Physical Activity [Steps], Exercise, and Education) eTable 1. Mean (SD) and Between-Group Differences in Primary Outcome at 3 Months for All Sensitivity Analyses eTable 2. Proportion of Participants Who Reported Improvement and Group Comparisons at 3-Month and 6-Month Follow-up for Disability Outcomes eTable 3. Odds Ratios for Secondary Binary Outcomes Comparing Groups at 3 Months and 6 Months eTable 4. Mean (SD) and Between-Group Differences for Exploratory Outcome at 3 Months and 6 Months eTable 5. Odds Ratios for Exploratory Binary Outcomes Comparing Groups at 3 Months and 6 Months [file jamanetwopen-e2320400-s002.pdf]

Supplementary Online Content

Marcuzzi A, Nordstoga AL, Bach K, et al. Effect of an artificial intelligence–based self-management app on musculoskeletal health in patients with neck and/or low back pain referred to specialist care: a randomized clinical trial. *JAMA Netw Open*. 2023;6(6):e2320400. doi:10.1001/jamanetworkopen.2023.20400

eFigure. Start Screen of the SELFBACK App (Left) and 3 Main Components of the Weekly Self-Management Plan (Physical Activity [Steps], Exercise, and Education)

eTable 1. Mean (SD) and Between-Group Differences in Primary Outcome at 3 Months for All Sensitivity Analyses

eTable 2. Proportion of Participants Who Reported Improvement and Group Comparisons at 3-Month and 6-Month Follow-up for Disability Outcomes

eTable 3. Odds Ratios for Secondary Binary Outcomes Comparing Groups at 3 Months and 6 Months

eTable 4. Mean (SD) and Between-Group Differences for Exploratory Outcome at 3 Months and 6 Months

eTable 5. Odds Ratios for Exploratory Binary Outcomes Comparing Groups at 3 Months and 6 Months

This supplemental material has been provided by the authors to give readers additional information about their work.

© 2023 Marcuzzi A et al. *JAMA Network Open*.

eFigure. Start Screen of the SELFBACK App (Left) and 3 Main Components of the Weekly Self-Management Plan (Physical Activity [Steps], Exercise, and Education)

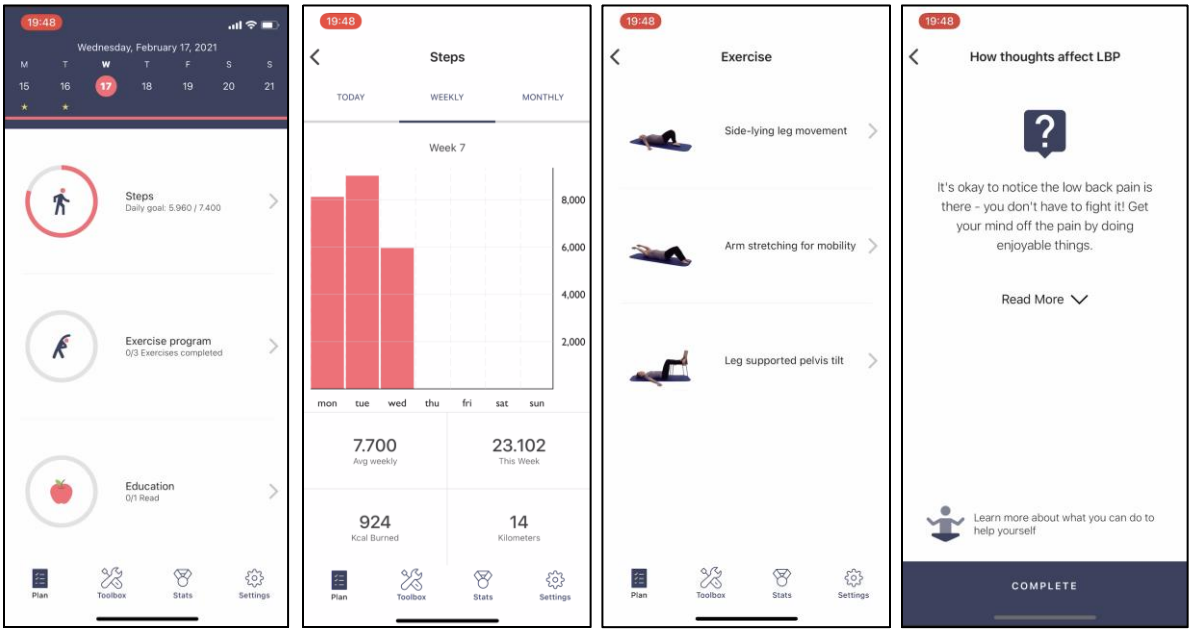

© 2023 Marcuzzi A et al. *JAMA Network Open*.

eTable 1. Mean (SD) and Between-Group Differences in Primary Outcome at 3 Months for All Sensitivity Analyses

	Mean (SD)[a]			SELFBACK vs Usual Care	SELFBACK vs e-Help
**Musculoskeletal Health Questionnaire**	**Usual Care (n = 97)**	**SELFBACK[b] (n = 99)**	**e-Help[c] (n = 98)**	**Between-group differences, mean (95% CI)[d]**	**Between-group differences, mean (95% CI)[d]**
Multiple imputation analyses (n=294)					
Baseline	29.2 (8.5)				
3 months	33.8 (10.1)	34.6 (10.2)	33.2 (11.0)	0.62 (-1.67 to 2.90)	1.08 (-1.24 to 3.41)
6 months	35.5 (10.4)	36.4 (9.9)	33.4 (10.5)	0.48 (-2.22 to 3.18)	2.44 (-0.25 to 5.12)
Complete case analyses (n=158)					
Baseline	30.4 (8.3)				
3 months	34.7 (9.1)	36.4 (9.3)	33.4 (11.0)	1.24 (-1.55 to 4.04)	2.44 (-0.38 to 5.26)
6 months	36.5 (10.5)	37.5 (9.9)	34.7 (10.3)	0.54 (-2.26 to 3.33)	2.12 (-0.70 to 4.94)
Per protocol analysis[e] (n=257)					
Baseline	29.0 (8.4)				
3 months	33.7 (10.1)	34.5 (10.0)	33.4 (10.8)	0.62 (-1.83 to 3.06)	0.84 (-1.68 to 3.35)
6 months	35.4 (10.4)	36.2 (10.2)	33.2 (10.5)	0.19 (-2.74 to 3.12)	2.16 (-0.78 to 5.11)
Per protocol analysis[f] (n=135)					
Baseline	29.5 (9.2)		---		
3 months	34.0 (10.1)	36.8 (9.3)	---	2.39 (-0.43 to 5.21)	---
6 months	35.6 (10.4)	37.5 (9.8)	---	1.09 (-2.25 to 4.44)	---

Abbreviations: SD, Standard Deviation; CI, Confidence Interval

[a] Marginal means from a crude linear mixed model and SDs from raw data among persons with information at the specific time points

[b] App-delivered self-management support in addition to usual care

[c] Web-based self-management support in addition to usual care

[d] Adjusted for age (years), sex (male, female), education (<10, 10-12, >12 years), average pain intensity past week at baseline (continuous, range 0-10)

[e] Adherence to the protocol was defined as accessing the intervention at least once for both intervention groups (SELFBACK group: n=70; e-Help group: n=90)

[f] Adherence to the protocol was defined as creating at least 6 weekly self-management plans during the first 12 weeks post randomization for SELFBACK group only (n=38)

© 2023 Marcuzzi A et al. *JAMA Network Open*.

eTable 2. Proportion of Participants Who Reported Improvement and Group Comparisons at 3-Month and 6-Month Follow-up for Disability Outcomes

	**Usual Care**		**SELFBACK[b]**		**e-Help[c]**		**SELFBACK vs Usual Care**	**SELFBACK vs e-Help**
	No. improved/ No. total	OR (95% CI)[a]	No. improved/ No. Total	OR (95% CI)[a]	No. improved/ No. Total	OR (95% CI)[a]	**Between-Group Differences, OR (95% CI)[a]**	**Between-Group Differences, OR (95% CI)[a]**
**Improvement in RMDQ & NDI combined[d]**								
Baseline	0/97	N/A	0/99	N/A	0/98	N/A	N/A	N/A
3 months	17/84	1.05 (0.60 to 1.84)	20/75	1.56 (0.74 to 3.31)	25/77	2.05 (0.98 to 4.26)	1.48 (0.70 to 3.14)	0.76 (0.38 to 1.52)
6 months	25/59	3.19 (1.62 to 6.26)	15/52	1.97 (0.88 to 4.41)	21/61	2.30 (1.05 to 5.00)	0.62 (0.28 to 1.34)	0.86 (0.39 to 1.89)

Abbreviations: CI, Confidence Interval; OR, Odds Ratio; RMDQ, Roland Morris Disability Questionnaire; NDI, Neck Disability Index; N/A, Not Applicable

[a] Adjusted for age (years), sex (male, female), education (<10, 10-12, >12 years), average pain intensity past week at baseline (continuous, range 0-10)

[b] App-delivered self-management support in addition to usual care

[c] Web-based self-management support in addition to usual care

[d] Improvement from baseline to follow up corresponds to a change of ≥4-points in RMDQ for people with low back pain only; or to a change of >7-point in NDI for people with neck pain only; or to either a change of ≥4-points in RMDQ or >7-point in NDI for people with both low back and neck pain.

© 2023 Marcuzzi A et al. *JAMA Network Open*.

eTable 3. Odds Ratios for Secondary Binary Outcomes Comparing Groups at 3 Months and 6 Months

	Usual Care		SELFBACK[b]		e-Help[c]		SELFBACK vs Usual Care	SELFBACK vs e-Help
	No. improved/ No. Total	OR (95% CI)[a]	No. improved/ No. Total	OR (95% CI)[a]	No. improved/ No. Total	OR (95% CI)[a]	**Between-group differences, OR (95% CI)[a]**	**Between-group differences, OR (95% CI)[a]**
**Average Pain Intensity Past Week, >5**								
Baseline, all groups	149/294	1.00 (reference)	---	---	---	---	---	---
3 months	29/85	0.52 (0.36 to 0.76)	25/76	0.52 (0.34 to 0.80)	32/77	0.74 (0.47 to 1.16)	1.01 (0.59 to 1.73)	0.71 (0.39 to 1.28)
6 months	21/60	0.55 (0.32 to 0.93)	14/53	0.45 (0.26 to 0.79)	27/62	0.69 (0.44 to 1.07)	0.83 (0.40 to 1.73)	0.66 (0.33 to 1.31)
**Worst Pain Intensity Past Week, >5**								
Baseline, all groups	241/294	1.00 (reference)	--	--	--	--	--	--
3 months	51/85	0.38 (0.24 to 0.60)	47/76	0.40 (0.26 to 0.62)	52/77	0.41 (0.25 to 0.69)	1.05 (0.60 to 1.83)	0.97 (0.53 to 1.76)
6 months	33/60	0 29 (0.17 to 0.51)	25/53	0.26 (0.15 to 0.44)	42/62	0.40 (0.24 to 0.67)	0.89 (0.45 to 1.78)	0.65 (0.34 to 1.28)
**Pain Self-Efficacy Questionnaire, high (>40)**								
Baseline, all groups	123/294	1.00 (reference)	--	--	--	--	--	--
3 months	48/84	1.94 (1.25 to 3.00)	48/75	2.28 (1.35 to 3.85)	37/77	1.31 (0.83 to 2.06)	1.18 (0.62 to 2.25)	1.74 (0.90 to 3.36)
6 months	35/59	2.41 (1.49 to 3.91)	33/52	1.57 (0.92 to 2.68)	29/59	1.14 (0.75 to 1.74)	0.65 (0.32 to 1.31)	1.37 (0.71 to 2.68)
**Brief Illness Perception Questionnaire, >75 perc. (>52)**								
Baseline, all groups	69/294	1.00 (reference)	--	--	--	--	--	--
3 months	12/84	0.60 (0.34 to 1.08)	13/75	0.72 (0.42 to 1.24)	19/77	1.15 (0.70 to 1.91)	1.19 (0.55 to 2.59)	0.62 (0.30 to 1.28)
6 months	7/58	0.53 (0.26 to 1.06)	6/52	0.48 (0.18 to 1.30)	18/59	1.53 (0.91 to 2.58)	0.92 (0.28 to 2.97)	0.32 (0.11 to 0.93)
**Global perceived effect, improved**								
Baseline, all groups	N/A		--	--	--	--	--	--
3 months	33/84	1.70 (1.06 to 2.72)	39/75	2.65 (1.36 to 5.16)	37/77	2.54 (1.31 to 4.94)	1.57 (0.83 to 2.94)	1.04 (0.55 to 1.99)
6 months	28/57	2.62 (1.44 to 4.76)	26/52	2.48 (1.19 to 5.15)	24/59	1.72 (0.85 to 5.15)	0.95 (0.45 to 2.00)	1.44 (0.68 to 3.04)

Abbreviations: CI, Confidence Interval; OR, odds ratio

[a] Adjusted for age (years), sex (male, female), education (<10, 10-12, >12 years), average pain intensity past week at baseline (continuous, range 0-10)

[b] App-delivered self-management support in addition to usual care

[c] Web-based self-management support in addition to usual care

© 2023 Marcuzzi A et al. *JAMA Network Open*.

eTable 4. Mean (SD) and Between-Group Differences for Exploratory Outcome at 3 Months and 6 Months

	Mean (SD)[a]			SELFBACK vs Usual Care	SELFBACK vs e-Help
	Usual Care (n = 98)	SELFBACK[b] (n = 99)	e-Help[c] (n = 97)	Between-group differences, mean (95% CI)[d]	Between-group differences, mean (95% CI)[d]
**Fear-Avoidance Belief Questionnaire (0-24)**					
Baseline	11.2 (6.1)				
3 months	9.1 (5.8)	8.8 (6.1)	8.8 (6.2)	-0.24 (-1.69 to 1.21)	0.16 (-1.32 to 1.63)
6 months	8.1 (5.3)	8.2 (5.4)	8.1 (5.4)	0.19 (-1.50 to 1.88)	0.33 (-1.35 to 2.02)
**Perceived Stress Scale (0-40)**					
Baseline	16.6 (7.0)				
3 months	16.7 (7.1)	14.8 (7.1)	16.0 (7.8)	-1.89 (-3.36 to -0.42)	-1.11 (-2.61 to 0.38)
6 months	15.5 (7.6)	14.7 (7.6)	15.7 (7.1)	-0.71 (-2.41 to 1.00)	-0.85 (-2.56 to 0.85)
**Patient Health Questionnaire-2 (0-6)**					
Baseline	1.9 (1.5)				
3 months	1.8 (1.4)	1.6 (1.2)	1.8 (1.7)	-0.24 (-0.59 to 0.10)	-0.16 (-0.52 to 0.19)
6 months	1.8 (1.4)	1.6 (1.5)	1.7 (1.4)	-0.17 (-0.58 to 0.23)	-0.06 (-0.46 to 0.34)
**Patient Specific Functional Scale (0-10)**					
Baseline	3.1 (2.2)				
3 months	4.4 (2.9)	4.1 (2.8)	4.1 (2.6)	-0.29 (-0.95 to 0.37)	-0.59 (-0.73 to 0.61)
6 months	5.0 (3.2)	4.4 (4.6)	4.4 (2.5)	-0.61 (-1.38 to 0.16)	-0.02 (-0.79 to 0.75)
**Work ability (0-10)**					
Baseline	4.5 (2.6)				
3 months	5.3 (2.4)	5.5 (2.4)	5.0 (2.6)	0.20 (-0.38 to 0.79)	0.45 (-0.14 to 1.05)
6 months	5.4 (2.7)	5.2 (2.6)	5.1 (2.4)	-0.24 (0.92 to 0.44)	0.07 (-0.61 to 0.75)

Abbreviations: SD, Standard Deviation; CI, Confidence Interval

[a] Marginal means from a crude linear mixed model and SDs from raw data among persons with information at the specific time points

[b] App-delivered self-management support in addition to usual care

[c] Web-based self-management support in addition to usual care

[d] Adjusted for age (years), sex (male, female), education (<10, 10-12, >12 years), average pain intensity past week at baseline (continuous, range 0-10)

© 2023 Marcuzzi A et al. *JAMA Network Open*.

eTable 5. Odds Ratios for Exploratory Binary Outcomes Comparing Groups at 3 Months and 6 Months

	Usual Care		SELFBACK[b]		e-Help[c]		SELFBACK vs Usual Care	SELFBACK vs e-Help
	No. improved/No. Total	OR (95% CI)[a]	No. improved/No. Total	OR (95% CI)[a]	No. improved/No. Total	OR (95% CI)[a]	**Between-group differences, OR (95% CI)[a]**	**Between-group differences, OR (95% CI)[a]**
**Fear-Avoidance Belief Questionnaire, >50 perc. (>11)**								
Baseline, all groups	145/294	1.00 (reference)						
3 months	29/84	0.47 (0.30 to 0.74)	25/75	0.49 (0.29 to 0.82)	30/77	0.65 (0.40 to 1.04)	1.04 (0.54 to 1.98)	0.76 (0.39 to 1.47)
6 months	17/59	0.34 (0.19 to 0.58)	15/52	0.44 (0.25 to 0.78)	17/60	0.43 (0.26 to 0.74)	1.30 (0.60 to 2.82)	1.01 (0.48 to 2.13)
**Perceived Stress Scale, high (>27)**								
Baseline, all groups	22/294	1.00 (reference)						
3 months	8/84	1.45 (0.61 to 3.47)	2/75	0.36 (0.11 to 1.13)	2/77	0.53 (0.21 to 1.35)	0.25 (0.06 to 1.06)	0.67 (0.15 to 2.95)
6 months	4/58	0.98 (0.35 to 2.78)	3/52	0.93 (0.19 to 4.42)	2/59	0.71 (0.32 to 1.58)	0.95 (0.16 to 5.72)	1.31 (0.24 to 7.14)
**Patient Health Questionnaire-2, ≥3**								
Baseline, all groups	82/294	1.00 (reference)						
3 months	20/84	0.86 (0.52 to 1.42)	15/75	0.71 (0.39 to 1.30)	18/77	0.91 (0.56 to 1.48)	0.82 (0.39 to 1.76)	0.78 (0.37 to 1.66)
6 months	15/57	0.94 (0.56 to 1.59)	9/52	0.81 (0.38 to 1.72)	11/59	0.71 (0.43 to 1.19)	0.85 (0.35 to 2.08)	1.13 (0.46 to 2.76)
**Saltin-Grimbv Physical Activity Level, moderate/vigorous**								
Baseline, all groups	84/294	1.00 (reference)						
3 months	22/84	0.81 (0.49 to 1.32)	22/75	0.98 (0.63 to 1.53)	23/77	1.28 (0.77 to 2.13)	1.21 (0.64 to 2.31)	0.77 (0.41 to 1.45)
6 months	20/59	1.29 (0.75 to 2.21)	13/52	0.76 (0.45 to 1.30)	22/60	1.64 (0.96 to 2.80)	0.59 (0.28 to 1.23)	0.47 (0.22 to 0.97)
**Sleep problems, insomnia**								
Baseline, all groups	140/294	1.00 (reference)						
3 months	32/84	0.71 (0.48 to 1.06)	25/75	0.56 (0.35 to 0.90)	23/77	0.51 (0.33 to 0.78)	0.79 (0.44 to 1.43)	1.11 (0.60 to 2.07)
6 months	22/59	0.64 (0.41 to 0.99)	13/52	0.41 (0.25 to 0.70)	16/60	0.54 (0.32 to 0.92)	0.65 (0.33 to 1.27)	0.76 (0.37 to 1.57)
**Work ability, high (>7)**								
Baseline, all groups	42/294	1.00 (reference)						
3 months	11/84	0.96 (0.51 to 1.80)	16/75	1.50 (0.82 to 2.75)	17/77	1.90 (1.01 to 3.56)	1.56 (0.70 to 3.46)	0.79 (0.35 to 1.76)
6 months	13/59	2.30 (1.19 to 4.44)	12/52	1.47 (0.72 to 3.01)	15/60	2.27 (1.11 to 4.63)	0.64 (0.26 to 1.59)	0.65 (0.25 to 1.66)
**Patient Acceptable Symptom State, acceptable**								
Baseline, all groups	N/A							
3 months	23/84	1.05 (0.64 to 1.73)	31/75	1.86 (0.94 to 3.68)	31/75	2.13 (1.07 to 4.25)	1.76 (0.90 to 3.44)	0.87 (0.44 to 1.71)
6 months	21/57	1.89 (0.97 to 3.71)	24/52	2.25 (1.07 to 4.71)	22/59	1.76 (0.86 to 3.62)	1.19 (0.54 to 2.61)	1.28 (0.60 to 2.72)

Abbreviations: CI, Confidence Interval; OR, odds ratio; N/A, Not Applicable
[a] Adjusted for age (years), sex (male, female), education (<10, 10-12, >12 years), average pain intensity past week at baseline (continuous, range 0-10)
[b] App-delivered self-management support in addition to usual care
[c] Web-based self-management support in addition to usual care

© 2023 Marcuzzi A et al. *JAMA Network Open*.
