# Supplementary material for: Effect of an Artificial Intelligence–Based Self-Management App on Musculoskeletal Health in Patients With Neck and/or Low Back Pain Referred to Specialist Care: A Randomized Clinical Trial
Source: JAMA Netw Open. 2023 Jun 27;6(6):e2320400. doi: 10.1001/jamanetworkopen.2023.20400 (PMC10300712; doi:10.1001/jamanetworkopen.2023.20400)
Supplement: Supplement 3. — Data Sharing Statement [file jamanetwopen-e2320400-s003.pdf]

Data Sharing Statement

Marcuzzi. Effect of an Artificial Intelligence–Based Self-Management App on Musculoskeletal Health in Patients With Neck and/or Low Back Pain Referred to Specialist Care. *JAMA Netw Open*. Published June 27, 2023. doi:10.1001/jamanetworkopen.2023.20400

Data
Data available: Yes
Data types: Other (please specify)
Additional Information: Data is kept for 5 years (reidentifiable data) and thereafter the data will be anonymised and stored up to 30 years.
How to access data: Contact to Paul Jarle Mork (paul.mork@ntnu.no)
When available: With publication

Supporting Documents
Document types: None

Additional Information
Who can access the data: Researchers whose proposed use of the data has been approved by the data steering group
Types of analyses: Any relevant research purpose
Mechanisms of data availability: After approval by the data steering group. Contact to Paul Jarle Mork (paul.mork@ntnu.no)
